# Supplementary material for: Plasma and Urinary Metabolomic Analysis of Gout and Asymptomatic Hyperuricemia and Profiling of Potential Biomarkers: A Pilot Study
Source: Biomedicines. 2024 Jan 27;12(2):300. doi: 10.3390/biomedicines12020300 (PMC10887286; doi:10.3390/biomedicines12020300)
Supplement: Supplementary file 1 [file biomedicines-12-00300-s001.zip › biomedicines-2820024-supplementary.pdf]

Table S1. Summary of metabolites detected by GC-MS/MS in this study.

Compound name [arb.unit]	Sample	Median (IQR: Q1-Q3)			Raw *P*
		All (n = 42)	AHU (n = 9)	Overall gout (n = 33)	
**2-Isopropylmalic acid-3TMS**	**Plasma**	**1**	**1**	**1**	**Reference**
1,5-Anhydro-glucitol-4TMS (×10^{-2})	Plasma	0.63 (0–1.00)	0.86 (0.67–1.48)	0.54 (0–0.95)	**0.037**
1,6-Anhydroglucose-3TMS (×10^{-2})	Plasma	0.73 (0–0.96)	0.63 (0–1.07)	0.83 (0–0.97)	0.741
1-Hexadecanol-TMS (×10^{-2})	Plasma	2.16 (0–2.66)	2.46 (2.09–2.53)	2.05 (1.63–2.62)	0.490
2-Aminoadipic acid-3TMS (×10^{-3})	Plasma	0 (0–9.49)	4.88 (0–8.35)	0 (0–10.47)	0.692
2-Aminobutyric acid-2TMS (×10^{-2})	Plasma	4.22 (3.29–5.73)	4.02 (3.77–5.79)	4.34 (3.23–5.92)	0.890
2-Aminoethanol-3TMS (×10^{-1})	Plasma	3.50 (3.00–4.04)	3.59 (2.96–4.29)	3.48 (3.00–4.05)	0.818
2-Aminoisobutyric acid-2TMS (×10^{-2})	Plasma	6.63 (5.56–7.38)	6.83 (5.26–8.02)	6.51 (5.57–7.40)	0.934
2-Aminopimelic acid-3TMS (×10^{-1})	Plasma	2.49 (2.13–2.80)	2.14 (1.97–2.48)	2.52 (2.34–2.82)	0.089
2-Deoxy-glucose-4TMS (×10^{-1})	Plasma	1.11 (0.86–1.37)	1.01 (0.83–1.27)	1.13 (0.87–1.41)	0.382
2-Hydroxybutyric acid-2TMS (×10^{-1})	Plasma	1.72 (1.35–2.30)	1.39 (1.09–2.02)	1.88 (1.48–2.31)	0.101
2-Hydroxyglutaric acid-3TMS (×10^{-2})	Plasma	1.32 (0.97–1.38)	1.14 (1.07–1.60)	1.42 (0.94–1.76)	0.988
2-Hydroxyisobutyric acid-2TMS (×10^{-2})	Plasma	1.09 (0.79–1.38)	0.93 (0.80–1.21)	1.14 (0.77–1.45)	0.399
2-Hydroxyisovaleric acid-2TMS (×10^{-2})	Plasma	3.78 (2.93–6.23)	3.55 (3.25–4.31)	4.17 (2.73–6.56)	0.613
2-Ketobutyric acid-meto-TMS (×10^{-2})	Plasma	2.47 (1.94–2.98)	2.14 (1.73–2.64)	2.54 (2.12–3.01)	0.137
2-Ketoglutaric acid-meto-2TMS (×10^{-2})	Plasma	7.04 (5.53–8.62)	5.18 (4.61–5.85)	7.33 (6.45–9.01)	**<0.001**
2-Ketoisocaproic acid-meto-TMS (×10^{-1})	Plasma	1.38 (1.11–1.64)	1.22 (1.17–1.60)	1.39 (1.08–1.70)	0.748
2-Keto-isovaleric acid-meto-TMS (×10^{-2})	Plasma	2.13 (1.66–2.55)	2.08 (1.86–2.43)	2.18 (1.62–2.72)	0.771
3-Aminoglutaric acid-2TMS (×10^{-2})	Plasma	1.72 (0–8.58)	6.97 (0.34–9.39)	1.35 (0–7.94)	0.260
3-Aminoisobutyric acid-3TMS (×10^{-3})	Plasma	0 (0–7.77)	0 (0–0)	0 (0–8.86)	0.082
3-Aminopropanoic acid-3TMS (×10^{-2})	Plasma	3.59 (1.19–7.08)	3.96 (2.28–5.78)	3.22 (0.57–7.84)	0.866
3-Hydroxybutyric acid-2TMS (×10^{-1})	Plasma	4.04 (3.26–6.16)	4.04 (2.67–5.06)	4.04 (3.37–6.75)	0.319
3-Hydroxyglutaric acid-3TMS (×10^{-1})	Plasma	8.01 (7.78–8.18)	8.03 (7.85–8.20)	7.97 (7.70–8.19)	0.613
3-Hydroxyisobutyric acid-2TMS (×10^{-1})	Plasma	1.90 (1.40–2.36)	1.84 (1.25–2.25)	1.93 (1.52–2.55)	0.263
3-Hydroxyisovaleric acid-2TMS (×10^{-2})	Plasma	4.75 (3.86–5.35)	4.79 (3.42–5.29)	4.71 (4.02–5.36)	0.382

3-Hydroxypropionic acid-2TMS (×10^{-2})	Plasma	7.08 (6.32–8.13)	6.45 (5.63–8.04)	7.38 (6.42–8.13)	0.334
3-Methyl-2-oxovaleric acid-meto-TMS (×10^{-1})	Plasma	1.24 (1.02–1.64)	1.22 (0.96–1.40)	1.30 (1.01–1.65)	0.434
3-Phenyllactic acid-2TMS (×10^{-3})	Plasma	2.62 (0–8.78)	0 (0–7.59)	5.36 (0–10.02)	0.219
4-Aminobutyric acid-3TMS	Plasma	0 (0–0)	0 (0–0)	0 (0–0)	0.935
4-Hydroxyphenyllactic acid-3TMS (×10^{-2})	Plasma	6.51 (4.68–8.55)	5.09 (4.14–7.47)	6.66 (4.91–8.84)	0.163
4-Hydroxyphenylpyruvic acid-meto-2TMS	Plasma	0 (0–0)	0 (0–0)	0 (0–0)	0.455
4-Hydroxyproline-3TMS (×10^{-1})	Plasma	1.61 (0.11–3.74)	3.15 (0.20–4.00)	0.80 (0.71–3.63)	0.365
5-Hydroxymethyl-2-furoic acid-2TMS	Plasma	0 (0–0)	0 (0–0)	0 (0–0)	0.602
5-Methoxytryptamine-2TMS (×10^{-2})	Plasma	1.52 (0.91–1.88)	1.65 (0.34–2.91)	1.44 (0.95–1.80)	0.724
5-Oxoproline-2TMS	Plasma	7.17 (5.85–7.84)	7.77 (6.71–8.75)	6.86 (5.72–7.71)	0.064
Acetoacetic acid-meto-TMS	Plasma	0 (0–0)	0 (0–0)	0 (0–0)	0.354
Acetylglycine-TMS	Plasma	0 (0–0)	0 (0–0)	0 (0–0)	0.279
Aconitic acid-3TMS (×10^{-3})	Plasma	0 (0–1.70)	0 (0–0)	0 (0–7.46)	0.065
Adipic acid-2TMS (×10^{-3})	Plasma	8.34 (4.69–11.11)	9.25 (2.48–12.48)	8.21 (4.66–11.11)	0.988
Alanine-2TMS	Plasma	24.1 (20.1–27.9)	24.4 (21.1–27.5)	23.8 (20.1–28.2)	0.635
Allose-meto-5TMS (×10^{-2})	Plasma	9.00 (6.37–11.04)	7.50 (5.78–9.90)	9.12 (6.42–11.49)	0.238
Arabitol-5TMS (×10^{-2})	Plasma	0.26 (0–1.15)	0 (0–0.88)	0.53 (0–1.26)	0.302
Arachidonic acid-TMS (×10^{-1})	Plasma	0.72 (0.52–0.86)	0.85 (0.73–1.02)	0.67 (0.43–0.83)	**0.015**
Arginine-3TMS (×10^{-2})	Plasma	1.95 (0.65–3.08)	2.19 (1.12–3.00)	1.83 (0.30–3.21)	0.634
Asparagine-3TMS (×10^{-2})	Plasma	1.43 (0–6.17)	6.38 (0–7.56)	1.31 (0–5.61)	0.380
Aspartic acid-3TMS (×10^{-2})	Plasma	1.31 (0–8.06)	6.46 (0–8.76)	1.08 (0–7.48)	0.322
Azelaic acid-2TMS	Plasma	0 (0–0)	0 (0–0)	0 (0–0)	0.787
Benzoic acid-TMS (×10^{-1})	Plasma	5.62 (4.84–6.62)	5.40 (4.68–6.27)	5.77 (4.91–7.08)	0.453
Caproic acid-TMS (×10^{-1})	Plasma	1.18 (1.03–1.39)	1.30 (1.07–1.41)	1.17 (0.95–1.41)	0.350
Cholesterol-TMS (×10^{-2})	Plasma	0.99 (0.71–1.28)	1.30 (0.79–1.51)	0.96 (0.68–1.12)	0.153
Citramalic acid-3TMS (×10^{-2})	Plasma	0.73 (0–1.06)	0.94 (0–1.21)	0.71 (0–1.00)	0.461
Citric acid-4TMS (×10^{-1})	Plasma	3.61 (3.10–4.07)	3.16 (2.94–3.79)	3.77 (3.35–4.15)	0.305
Citrulline-3TMS	Plasma	0 (0–0)	0 (0–0)	0 (0–0)	0.602
Creatinine-3TMS (×10^{-3})	Plasma	0 (0–5.41)	0 (0–6.63)	0 (0–5.16)	0.926

Cystamine-nTMS	Plasma	0 (0–0)	0 (0–0)	0 (0–0)	0.602
Cysteine-3TMS (×10^{-2})	Plasma	2.21 (0–8.53)	7.64 (0.31–11.68)	1.93 (0–7.40)	0.115
Cystine-4TMS (×10^{-2})	Plasma	3.12 (2.46–4.51)	2.58 (1.62–3.01)	3.22 (2.59–5.06)	0.055
Decanoic acid-TMS (×10^{-2})	Plasma	6.69 (5.63–7.34)	6.66 (6.02–7.09)	6.74 (5.25–7.46)	0.915
Docosahexaenoic acid-TMS (×10^{-2})	Plasma	1.20 (0–1.78)	0.55 (0–1.45)	1.25 (0.38–1.93)	0.117
Eicosapentaenoic acid-TMS (×10^{-2})	Plasma	7.33 (5.33–8.83)	9.31 (7.38–9.53)	7.18 (4.92–8.55)	**0.013**
Elaidic acid-TMS (×10^{-2})	Plasma	5.92 (4.74–7.34)	4.99 (3.78–6.12)	6.61 (5.02–7.98)	**0.033**
Fructose-meto-5TMS (×10^{-2})	Plasma	2.04 (1.32–4.26)	2.36 (1.81–5.55)	1.97 (1.22–2.57)	0.125
Fumaric acid-2TMS (×10^{-2})	Plasma	1.98 (1.74–2.40)	1.82 (1.50–2.27)	2.02 (1.83–2.47)	0.129
Galacturonic acid-meto-5TMS (×10^{-2})	Plasma	0 (0–1.00)	0 (0–0.74)	0 (0–1.25)	0.386
Glucaric acid-6TMS (×10^{-2})	Plasma	1.02 (0.69–1.34)	0.91 (0.32–1.13)	1.04 (0.81–1.44)	0.136
Gluconic acid-6TMS (×10^{-2})	Plasma	3.42 (2.77–4.26)	3.58 (2.80–4.27)	3.42 (2.73–4.29)	0.939
Glucosamine-5TMS (×10^{-2})	Plasma	1.86 (1.31–2.30)	1.85 (1.60–2.23)	1.90 (1.10–2.31)	0.701
Glucose-meto-5TMS	Plasma	7.02 (5.98–8.67)	5.88 (4.93–6.75)	7.43 (6.35–9.07)	**0.006**
Glucuronic acid-meto-5TMS (×10^{-2})	Plasma	1.90 (1.39–2.71)	1.73 (1.10–2.16)	2.01 (1.59–2.99)	0.203
Glutamic acid-3TMS (×10^{-1})	Plasma	2.80 (2.37–5.87)	4.59 (0.58–7.09)	1.70 (0.22–4.90)	0.101
Glutamine-3TMS (×10^{-1})	Plasma	4.82 (1.28–8.37)	7.33 (1.71–9.59)	4.74 (1.23–8.36)	0.305
Glutaric acid-2TMS (×10^{-2})	Plasma	1.62 (1.16–2.02)	1.65 (1.35–1.76)	1.56 (1.16–2.06)	0.818
Glyceric acid-3TMS (×10^{-1})	Plasma	1.27 (1.10–1.56)	1.28 (1.21–1.68)	1.27 (1.10–1.56)	0.530
Glycerol 3-phosphate-4TMS (×10^{-2})	Plasma	0 (0–1.25)	0 (0–0.94)	0.95 (0–1.29)	0.109
Glycerol-3TMS (×10^{-1})	Plasma	1.00 (0.80–1.25)	1.06 (0.79–1.23)	0.99 (0.80–1.34)	0.988
Glycine-3TMS	Plasma	4.87 (3.25–5.88)	5.92 (4.09–7.49)	4.81 (3.07–5.66)	0.101
Glycolic acid-2TMS (×10^{-1})	Plasma	2.15 (1.93–2.39)	2.19 (1.91–2.45)	2.12 (1.92–2.37)	0.771
Glyoxylic acid-meto-TMS (×10^{-1})	Plasma	4.95 (4.64–5.40)	5.06 (4.73–5.31)	4.93 (4.47–5.66)	0.963
Histidine-3TMS (×10^{-1})	Plasma	1.50 (0.54–2.76)	1.64 (0.93–2.91)	1.24 (0.43–2.91)	0.350
Homocysteine-3TMS	Plasma	0 (0–0)	0 (0–0)	0 (0–0)	0.602
Hydroxylamine-3TMS (×10^{-1})	Plasma	6.39 (5.50–8.19)	6.24 (4.48–6.86)	7.32 (5.60–8.45)	0.154
Hypotaurine-3TMS (×10^{-2})	Plasma	0.82 (0–1.55)	0.84 (0–1.11)	0.81 (0–1.61)	0.646
Hypoxanthine-2TMS (×10^{-2})	Plasma	0 (0–0.81)	0 (0–1.18)	0 (0–0.79)	0.488

Indol-3-acetic acid-2TMS ($\times10^{-2}$)	Plasma	3.32 (2.11–3.90)	3.89 (3.10–5.10)	3.15 (2.00–3.61)	0.078
Inositol-6TMS ($\times10^{-1}$)	Plasma	1.43 (1.11–1.83)	1.31 (0.99–1.75)	1.49 (1.11–1.92)	0.453
Isocitric acid-4TMS ($\times10^{-2}$)	Plasma	2.18 (1.96–2.73)	1.89 (1.62–2.13)	2.34 (2.07–2.86)	**0.015**
Isoleucine-2TMS	Plasma	2.89 (1.61–3.63)	3.39 (2.38–3.53)	2.87 (1.43–3.73)	0.366
Kynurenine-3TMS ($\times10^{-3}$)	Plasma	0 (0–5.11)	0 (0–5.36)	0 (0–4.60)	0.985
Lactic acid-2TMS	Plasma	7.42 (6.38–9.41)	6.28 (5.63–7.71)	7.86 (6.77–10.00)	**0.018**
Lactose-meto-8TMS	Plasma	0 (0–0)	0 (0–0)	0 (0–0)	0.602
Lauric acid-TMS ($\times10^{-1}$)	Plasma	0.88 (0.71–1.03)	0.99 (0.89–1.03)	0.83 (0.67–1.06)	0.137
Leucine-2TMS	Plasma	5.56 (3.07–6.85)	6.85 (4.59–7.24)	5.39 (2.72–6.28)	0.083
Linoleic acid-TMS ($\times10^{-1}$)	Plasma	1.51 (1.19–1.86)	1.44 (1.11–1.68)	1.53 (1.24–1.92)	0.471
Lysine-4TMS	Plasma	0.57 (0.36–0.86)	0.59 (0.51–0.97)	0.55 (0.35–0.81)	0.319
Lyxose-meto-4TMS	Plasma	0 (0–0)	0 (0–0)	0 (0–0)	0.602
Maleic acid-2TMS	Plasma	0 (0–0)	0 (0–0)	0 (0–0)	0.860
Malic acid-3TMS ($\times10^{-3}$)	Plasma	5.02 (0–6.70)	4.62 (0–5.33)	5.51 (0–7.05)	0.349
Mannitol-6TMS	Plasma	1.04 (0.87–1.26)	0.94 (0.78–1.03)	1.10 (0.91–1.35)	**0.033**
Mannose-meto-5TMS ($\times10^{-1}$)	Plasma	1.23 (0.92–1.43)	0.93 (0.85–1.37)	1.24 (1.03–1.60)	0.095
Margaric acid-TMS ($\times10^{-2}$)	Plasma	4.10 (3.62–4.88)	4.39 (3.78–5.08)	4.01 (3.51–4.85)	0.434
meso-Erythritol-4TMS ($\times10^{-3}$)	Plasma	0 (0–0)	0 (0–0)	0 (0–0)	0.174
Methionine-2TMS ($\times10^{-2}$)	Plasma	0 (0–2.66)	2.39 (0–3.73)	0 (0–2.53)	0.343
Myristic acid-TMS ($\times10^{-1}$)	Plasma	1.53 (1.26–1.82)	1.70 (1.44–1.85)	1.50 (1.16–1.80)	0.193
N6-Acetyllysine-2TMS	Plasma	0 (0–0)	0 (0–0)	0 (0–0)	0.602
N-Acetylglutamine-3TMS	Plasma	0 (0–0)	0 (0–0)	0 (0–0)	0.279
Niacinamide-TMS	Plasma	0 (0–0)	0 (0–0)	0 (0–0)	0.602
Nicotinic acid-TMS	Plasma	0 (0–0)	0 (0–0)	0 (0–0)	0.833
Nonanoic acid-TMS ($\times10^{-1}$)	Plasma	3.63 (2.98–6.88)	6.84 (6.27–7.29)	3.31 (2.67–5.93)	**0.004**
Octadecanol-TMS ($\times10^{-2}$)	Plasma	8.08 (7.18–9.13)	7.53 (7.22–8.30)	8.15 (6.98–9.25)	0.193
Octanoic acid-TMS ($\times10^{-2}$)	Plasma	6.20 (5.09–6.65)	6.29 (5.78–6.65)	5.83 (4.70–6.65)	0.305
Oleamide-TMS ($\times10^{-1}$)	Plasma	2.58 (2.10–3.82)	2.74 (2.08–3.88)	2.58 (2.14–3.85)	0.818
Oleic acid-TMS ($\times10^{-1}$)	Plasma	4.75 (3.55–5.52)	4.17 (3.06–5.09)	4.76 (3.60–5.75)	0.250

O-Phosphoethanolamine-3TMS (×10^{-3})	Plasma	0 (0–0)	0 (0–0)	0 (0–0)	0.821
Ornithine-4TMS	Plasma	1.37 (0.57–1.85)	1.61 (0.75–1.85)	1.33 (0.54–1.90)	0.510
Oxalic acid-2TMS	Plasma	3.60 (3.09–4.09)	3.94 (3.60–4.11)	3.48 (2.97–4.09)	0.214
Palmitic acid-TMS	Plasma	4.77 (3.97–5.61)	5.32 (4.38–5.64)	4.59 (3.96–5.62)	0.471
Palmitoleic acid-TMS (×10^{-2})	Plasma	4.24 (2.49–5.64)	2.55 (2.05–4.64)	4.59 (2.96–5.80)	0.089
ParaXanthine-TMS	Plasma	0 (0–0)	0 (0–0)	0 (0–0)	0.480
Phenylacetic acid-TMS	Plasma	0 (0–0)	0 (0–0)	0 (0–0)	0.279
Phenylalanine-2TMS	Plasma	0.47 (0.07–2.31)	1.75 (0.09–2.57)	0.40 (0.68–2.30)	0.350
Phosphoric acid-3TMS	Plasma	20.8 (19.0–24.3)	23.9 (20.6–27.6)	20.2 (18.2–22.5)	**0.024**
Proline-2TMS	Plasma	2.13 (1.25–4.06)	2.64 (1.92–4.42)	1.83 (1.22–3.98)	0.203
Psicose-meto-5TMS (×10^{-2})	Plasma	1.90 (1.27–4.20)	3.16 (1.79–5.00)	1.84 (0.93–3.37)	0.078
Putrescine-4TMS (×10^{-2})	Plasma	0 (0–1.67)	0 (0–1.71)	0 (0–1.67)	0.854
Pyrogallol-3TMS (×10^{-3})	Plasma	0 (0–0)	0 (0–5.30)	0 (0–0)	**0.006**
Pyruvic acid-meto-TMS	Plasma	0.67 (0.57–0.81)	0.60 (0.54–0.67)	0.70 (0.60–0.91)	**0.048**
Ribitol-5TMS (×10^{-3})	Plasma	4.33 (0–9.52)	0 (0–6.82)	5.90 (0–9.61)	0.163
Ribonic acid-5TMS (×10^{-2})	Plasma	1.93 (0–2.69)	0 (0–2.04)	2.22 (0–2.83)	0.170
Serine-3TMS	Plasma	0.48 (0.19–1.02)	0.98 (0.46–1.12)	4.24 (1.81–8.68)	0.083
Sorbitol-6TMS (×10^{-1})	Plasma	1.31 (0.94–1.97)	1.95 (0.81–2.40)	1.26 (0.95–1.90)	0.305
Sorbose-meto-5TMS (×10^{-2})	Plasma	1.14 (0–2.18)	2.14 (0.61–4.31)	0.98 (0–1.89)	**0.043**
Stearic acid-TMS	Plasma	3.36 (2.93–3.86)	3.70 (3.34–4.06)	3.29 (2.88–3.71)	0.137
Succinic acid-2TMS (×10^{-1})	Plasma	0.87 (0.80–0.96)	0.86 (0.83–0.97)	0.88 (0.78–0.96)	0.725
Sucrose-8TMS (×10^{-2})	Plasma	0 (0–1.24)	1.69 (0–6.92)	0 (0–0.79)	**0.029**
Tagatose-meto-5TMS (×10^{-2})	Plasma	1.14 (0–2.99)	2.12 (0–5.66)	0.83 (0–1.57)	0.165
Tartaric acid-4TMS (×10^{-3})	Plasma	0 (0–0)	0 (0–0)	0 (0–0)	0.174
Taurine-3TMS (×10^{-2})	Plasma	1.33 (0.32–2.13)	1.16 (0.50–2.54)	1.37 (0.21–2.19)	0.841
Threitol-4TMS	Plasma	0 (0–0)	0 (0–0)	0 (0–0)	0.455
Threonic acid-4TMS (×10^{-2})	Plasma	3.93 (2.68–4.44)	3.63 (2.60–4.46)	3.97 (2.57–4.53)	0.939
Threonine-3TMS (×10^{-1})	Plasma	3.80 (1.50–6.70)	6.18 (3.43–6.73)	3.06 (1.37–6.36)	0.182
Triethanolamine-3TMS (×10^{-2})	Plasma	1.59 (1.16–2.13)	1.58 (1.10–2.90)	1.60 (1.16–2.10)	0.771

Tryptophan-3TMS	Plasma	1.22 (0.93–1.74)	1.58 (1.19–2.20)	1.11 (0.85–1.70)	0.055
Tyramine-3TMS	Plasma	1.20 (0.71–1.67)	1.27 (0.97–1.91)	1.15 (0.68–1.63)	0.366
Tyrosine-3TMS	Plasma	3.35 (2.33–4.74)	3.44 (2.98–4.46)	3.10 (2.16–4.89)	0.490
Urea-2TMS	Plasma	9.39 (7.79–12.19)	10.81 (8.43–11.52)	8.95 (7.67–12.44)	0.453
Uric acid-4TMS	Plasma	1.59 (1.46–1.92)	1.46 (1.39–1.78)	1.60 (1.51–1.95)	0.173
Valine-2TMS	Plasma	13.7 (11.0–16.2)	15.5 (13.6–16.6)	12.6 (10.3–16.3)	0.154
Xanthine-3TMS	Plasma	0 (0–0)	0 (0–0)	0 (0–0)	0.602
Xylitol-5TMS	Plasma	0 (0–0)	0 (0–0)	0 (0–0)	0.602
Xylose-meto-4TMS	Plasma	0 (0–0)	0 (0–0)	0 (0–0)	0.455
**2-Isopropylmalic acid-3TMS**	**Urinary**	**1**	**1**	**1**	**Reference**
1,6-Anhydroglucose-3TMS ($\times10^{-1}$)	Urinary	2.73 (1.19–3.61)	2.17 (0.59–5.83)	2.75 (1.33–3.56)	0.890
1-Hexadecanol-TMS ($\times10^{-1}$)	Urinary	1.80 (1.46–2.13)	1.93 (1.56–2.27)	1.77 (1.41–2.04)	0.305
2-Aminobutyric acid-2TMS ($\times10^{-2}$)	Urinary	1.17 (0.66–1.70)	1.28 (1.13–1.59)	1.05 (0.61–1.82)	0.350
2-Aminoethanol-3TMS	Urinary	11.01 (7.71–18.13)	14.79 (8.73–20.02)	10.84 (6.84–17.62)	0.490
2-Aminopimelic acid-3TMS	Urinary	8.69 (6.25–13.99)	10.16 (5.04–15.38)	8.44 (6.12–13.23)	0.748
2-Hydroxybutyric acid-2TMS	Urinary	0 (0–0)	0 (0–0)	0 (0–0)	0.354
2-Hydroxyglutaric acid-3TMS ($\times10^{-1}$)	Urinary	4.98 (3.22–8.02)	5.90 (1.94–8.78)	4.46 (3.31–7.56)	0.794
2-Hydroxyhippuric acid-2TMS ($\times10^{-2}$)	Urinary	3.42 (1.43–6.91)	4.72 (1.98–11.03)	2.79 (1.42–6.32)	0.391
2-Hydroxyisobutyric acid-2TMS ($\times10^{-1}$)	Urinary	1.25 (0.89–1.76)	1.28 (0.67–2.07)	1.22 (0.90–1.71)	0.890
2-Hydroxyisovaleric acid-2TMS	Urinary	0 (0–0)	0 (0–0)	0 (0–0)	0.220
2-Ketoadipic acid-oxime-3TMS	Urinary	0 (0–0)	0 (0–0)	0 (0–0)	0.455
2-Ketobutyric acid-meto-TMS ($\times10^{-2}$)	Urinary	1.60 (1.24–1.89)	1.38 (1.13–1.79)	1.62 (1.23–1.92)	0.173
2-Ketoglutaric acid-meto-2TMS	Urinary	0.93 (0.54–1.29)	1.17 (0.29–1.54)	0.90 (0.56–1.20)	0.988
2-Ketoisocaproic acid-meto-TMS ($\times10^{-3}$)	Urinary	0 (0–9.71)	6.61 (0–9.61)	0 (0–10.21)	0.750
2-Keto-isovaleric acid-meto-TMS	Urinary	0 (0–0)	0 (0–0)	0 (0–0)	0.354
2-Methyl-3-hydroxybutyric acid-2TMS ($\times10^{-3}$)	Urinary	0 (0–1.61)	0 (0–0)	0 (0–6.55)	0.356
2-Phosphoglyceric acid -4TMS ($\times10^{-3}$)	Urinary	0 (0–0)	0 (0–6.60)	0 (0–0)	0.125
3-Aminoglutaric acid-2TMS ($\times10^{-2}$)	Urinary	4.97 (3.03–8.78)	7.64 (5.35–10.38)	4.31 (2.84–8.18)	0.095
3-Aminoisobutyric acid-3TMS ($\times10^{-1}$)	Urinary	2.31 (0.60–11.10)	1.31 (0.79–15.74)	2.84 (0.55–1.16)	0.988
3-Aminopropanoic acid-3TMS ($\times10^{-1}$)	Urinary	0.82 (0.40–2.39)	0.80 (0.32–1.18)	0.82 (0.41–2.94)	0.434

3-Hydroxy-3-methylglutaric acid-3TMS (×10^{-2})	Urinary	5.34 (3.98–7.42)	6.68 (2.04–7.69)	5.24 (4.14–7.33)	0.842
3-Hydroxyanthranilic acid-3TMS (×10^{-3})	Urinary	0 (0–8.61)	0 (0–2.72)	0 (0–1.01)	0.443
3-Hydroxybutyric acid-2TMS (×10^{-1})	Urinary	1.96 (1.61–2.35)	1.75 (1.35–2.15)	2.01 (1.62–2.40)	0.089
3-Hydroxyglutaric acid-3TMS (×10^{-1})	Urinary	7.93 (7.58–8.26)	8.12 (7.93–8.23)	7.83 (7.52–8.30)	0.214
3-Hydroxyisobutyric acid-2TMS (×10^{-1})	Urinary	1.65 (1.20–2.13)	1.79 (1.01–2.05)	1.63 (1.25– 2.21)	0.725
3-Hydroxyisovaleric acid-2TMS (×10^{-1})	Urinary	4.10 (3.10–6.28)	4.64 (2.80–5.33)	4.02 (3.08–7.20)	0.657
3-Hydroxyphenylacetic acid-2TMS (×10^{-2})	Urinary	0 (0–0.91)	0 (0–1.15)	0 (0–0.93)	0.623
3-Hydroxypropionic acid-2TMS (×10^{-1})	Urinary	1.22 (0.83–2.02)	1.31 (0.77–2.25)	1.18 (0.87–1.74)	0.842
3-Methoxy-4-hydroxybenzoic acid-2TMS (×10^{-2})	Urinary	1.06 (0–1.80)	0.77 (0–1.08)	1.29 (0–1.95)	0.094
3-Methyl-2-oxovaleric acid-meto-TMS (×10^{-2})	Urinary	1.49 (0.64–1.95)	0.88 (0.64–1.70)	1.51 (0.72–2.08)	0.499
3-Methyladipic acid-2TMS (×10^{-2})	Urinary	2.68 (0–5.85)	0 (0–3.04)	2.84 (0–6.42)	0.175
3-Methylglutaric acid-2TMS (×10^{-2})	Urinary	1.52 (1.01–2.51)	1.48 (1.15–2.23)	1.55 (0.47–2.89)	0.939
3-Phenyllactic acid-2TMS (×10^{-2})	Urinary	0.24 (0–1.40)	0 (0–0.56)	0.62 (0–1.58)	0.185
3-Phosphoglyceric acid-4TMS (×10^{-3})	Urinary	0 (0–9.87)	7.35 (0–18.50)	0 (0–8.69)	0.312
3-Sulfinoalanine-3TMS (×10^{-3})	Urinary	0 (0–3.87)	0 (0–4.51)	0 (0–1.89)	0.318
4-Aminobutyric acid-3TMS (×10^{-2})	Urinary	4.24 (0–7.43)	7.35 (3.82–9.97)	3.63 (0–6.28)	0.051
4-Hydroxybenzoic acid-2TMS (×10^{-2})	Urinary	3.10 (1.87–3.89)	2.70 (1.72–3.99)	3.10 (1.99–3.95)	0.890
4-Hydroxyphenylacetic acid-2TMS (×10^{-1})	Urinary	0.85 (0.35–1.49)	0.93 (0.29–1.51)	7.94 (3.92–1.51)	0.657
4-Hydroxyphenyllactic acid-3TMS (×10^{-1})	Urinary	1.29 (0.73–2.24)	1.32 (0.41–1.78)	1.27 (0.80–2.45)	0.510
4-Hydroxyproline-3TMS (×10^{-2})	Urinary	0 (0–5.24)	0 (0–9.19)	0 (0–5.17)	0.986
5-Aminolevulinic acid-meto-3TMS (×10^{-2})	Urinary	1.33 (0.87–1.82)	1.72 (1.14–2.39)	1.10 (0.42–1.62)	0.106
5-Aminovaleric acid-3TMS (×10^{-1})	Urinary	0.72 (0.53–1.32)	0.76 (0.52–1.33)	0.70 (0.52–1.24)	0.657
5-Hydroxymethyl-2-furoic acid-2TMS (×10^{-1})	Urinary	1.50 (0.67–2.24)	1.84 (0.42–2.66)	1.49 (0.66–2.13)	0.842
5-Oxoproline-2TMS	Urinary	9.20 (7.40–11.82)	10.09 (6.18–11.52)	8.49 (7.48–11.85)	0.963
7-Methylguanine-2TMS (×10^{-1})	Urinary	3.63 (2.25–5.57)	4.00 (2.32–8.43)	3.23 (2.24–5.37)	0.226
Acetoacetic acid-meto-TMS	Urinary	0 (0–0)	0 (0–0)	0 (0–0)	0.602
Acetylglycine-TMS	Urinary	0 (0–0)	0 (0–0)	0 (0–0)	0.354
Aconitic acid-3TMS	Urinary	1.64 (0.98–2.71)	2.63 (0.89–2.92)	1.59 (0.98–2.47)	0.530
Adenine-2TMS (×10^{-1})	Urinary	1.60 (1.18–2.45)	1.72 (1.17–4.07)	1.57 (1.13–2.30)	0.382

Adenosine-4TMS (×10^{-1})	Urinary	0.91 (0.67–1.51)	1.31 (0.78–2.39)	0.90 (0.61–1.28)	0.108
Adipic acid-2TMS (×10^{-2})	Urinary	1.70 (1.07–2.85)	1.32 (0.78–1.97)	2.12 (1.10–3.07)	0.163
Alanine-2TMS	Urinary	10.44 (6.52–14.10)	12.72 (8.70–13.82)	10.29 (6.15–14.53)	0.571
Allantoin-3TMS (×10^{-2})	Urinary	4.54 (1.74–9.02)	4.74 (0.84–1.11)	4.35 (1.84–8.63)	0.939
Allose-meto-5TMS (×10^{-2})	Urinary	7.20 (4.96–9.53)	7.43 (4.22–24.13)	6.72 (4.92–9.24)	0.350
Arabinose-meto-4TMS (×10^{-1})	Urinary	3.40 (2.44–5.35)	3.98 (2.15–6.26)	3.19 (2.40–5.20)	0.434
Arabitol-5TMS (×10^{-1})	Urinary	6.78 (4.37–9.89)	8.37 (4.25–8.74)	6.09 (4.37–10.54)	0.939
Arachidonic acid-TMS	Urinary	0 (0–0)	0 (0–0)	0 (0–0)	0.602
Arginine-3TMS (×10^{-1})	Urinary	1.15 (0.40–1.75)	1.51 (1.14–2.33)	0.89 (0.33–1.61)	0.145
Ascorbic acid-4TMS	Urinary	0 (0–0)	0 (0–0)	0 (0–0)	0.928
Asparagine-3TMS (×10^{-1})	Urinary	1.27 (0.69–3.31)	1.14 (0–3.21)	1.30 (0.83–3.32)	0.283
Aspartic acid-3TMS (×10^{-2})	Urinary	4.35 (2.59–7.91)	6.32 (4.82–9.39)	3.85 (2.47–7.50)	0.122
Azelaic acid-2TMS (×10^{-2})	Urinary	0.91 (0–2.98)	0 (0–0.36)	1.33 (0–3.19)	**0.030**
Benzoic acid-TMS (×10^{-1})	Urinary	4.43 (3.49–4.93)	4.45 (3.68–4.80)	4.42 (3.43–5.04)	0.657
Caproic acid-TMS (×10^{-1})	Urinary	1.09 (0.96–1.24)	1.13 (0.93–1.33)	1.07 (0.96–1.25)	0.988
Catechol-2TMS (×10^{-2})	Urinary	3.13 (1.28–6.16)	3.18 (2.16–8.76)	2.14 (1.15–5.84)	0.444
Cholesterol-TMS	Urinary	0 (0–0)	0 (0–0)	0 (0–0)	0.602
Citramalic acid-3TMS (×10^{-1})	Urinary	3.91 (2.82–5.41)	3.88 (2.20–9.98)	3.95 (2.75–5.23)	0.657
Citric acid-4TMS	Urinary	13.15 (9.33–20.96)	14.94 (7.67–23.22)	13.02 (9.29–19.21)	0.748
Creatinine-3TMS	Urinary	0.65 (0.41–1.03)	1.02 (0.53–2.00)	0.60 (0.40–0.91)	0.055
Cystathionine-4TMS (×10^{-2})	Urinary	0.11 (0–1.63)	0 (0–0.94)	0.67 (0–1.74)	0.258
Cysteine-3TMS (×10^{-1})	Urinary	2.13 (1.18–3.62)	2.75 (1.64–4.74)	1.94 (1.09–3.36)	0.238
Cystine-4TMS (×10^{-2})	Urinary	3.55 (1.42–5.48)	3.64 (3.04–4.43)	2.74 (1.16–7.10)	0.434
Cytosine-2TMS (×10^{-1})	Urinary	1.42 (0.95–2.08)	1.79 (0.66–2.67)	1.40 (0.94–2.04)	0.635
Decanoic acid-TMS (×10^{-2})	Urinary	5.40 (4.59–6.54)	5.98 (4.55–7.39)	5.33 (4.67–6.51)	0.471
Dihydroxyacetone phosphate-meto-3TMS (×10^{-1})	Urinary	1.12 (0.57–1.71)	1.52 (0.63–1.94)	1.02 (0.57–1.60)	0.382
Docosahexaenoic acid-TMS (×10^{-2})	Urinary	0 (0–1.17)	0 (0–1.40)	0 (0–1.16)	0.919
Elaidic acid-TMS (×10^{-1})	Urinary	0.94 (0.61–1.35)	0.94 (0.87–1.20)	0.83 (0.59–1.65)	0.417
Ethylmalonic acid-2TMS (×10^{-1})	Urinary	0.82 (0.50–1.18)	0.96 (0.46–1.11)	0.82 (0.53–1.19)	0.939

Fructose-meto-5TMS (×10^{-2})	Urinary	4.08 (1.85–8.31)	7.50 (1.47–15.30)	3.82 (1.93–7.46)	0.263
Fucose-meto-4TMS (×10^{-2})	Urinary	0 (0–1.53)	0 (0–1.89)	0 (0–1.54)	0.696
Fumaric acid-2TMS (×10^{-2})	Urinary	5.95 (4.17–7.69)	5.23 (3.05–5.98)	6.54 (4.52–7.78)	0.122
Galactitol-6TMS (×10^{-1})	Urinary	1.49 (0.79–2.44)	1.64 (1.15–4.71)	1.40 (0.78–2.37)	0.214
Galacturonic acid-meto-5TMS	Urinary	1.20 (0.85–1.83)	1.56 (0.74–2.55)	1.14 (0.94–1.79)	0.490
Glucaric acid-6TMS	Urinary	1.11 (0.84–1.62)	1.45 (0.86–2.13)	1.09 (0.83–1.48)	0.366
Gluconic acid-6TMS	Urinary	2.93 (1.95–4.29)	5.08 (1.93–5.51)	2.82 (1.88–3.90)	0.114
Glucosamine-5TMS (×10^{-1})	Urinary	3.40 (2.20–6.34)	5.78 (3.13–6.95)	3.35 (2.19–6.25)	0.290
Glucose 6-phosphate-meto-6TMS	Urinary	0 (0–0)	0 (0–0)	0 (0–0)	0.602
Glucose-meto-5TMS (×10^{-1})	Urinary	4.57 (3.21–7.76)	5.20 (2.60–8.50)	4.38 (3.25–7.08)	0.963
Glucuronic acid-meto-5TMS	Urinary	3.27 (2.04–5.19)	3.83 (1.57–5.51)	3.19 (2.18–4.00)	0.818
Glutamic acid-3TMS (×10^{-1})	Urinary	1.84 (1.34–2.74)	2.72 (1.44–3.29)	1.70 (1.32–2.60)	0.214
Glutamine-3TMS (×10^{-1})	Urinary	2.54 (1.14–5.11)	3.65 (2.60–6.14)	2.34 (1.08–5.20)	0.214
Glyceric acid-3TMS (×10^{-1})	Urinary	1.68 (0.99–2.30)	1.50 (0.82–1.89)	1.93 (1.06–2.38)	0.238
Glycerol 3-phosphate-4TMS (×10^{-1})	Urinary	2.66 (1.47–4.08)	3.70 (1.68–4.43)	2.51 (1.46–3.86)	0.382
Glycine-3TMS	Urinary	11.93 (8.14–25.81)	17.50 (12.37–28.87)	10.27 (7.78–20.42)	0.122
Glycolic acid-2TMS	Urinary	1.24 (0.93–1.85)	1.48 (0.87–2.38)	1.22 (0.92–1.81)	0.490
Glycyl-Glycine-4TMS	Urinary	0 (0–0)	0 (0–0)	0 (0–0)	0.056
Guanine-3TMS (×10^{-2})	Urinary	6.55 (3.14–9.89)	7.87 (4.98–11.56)	6.05 (2.38–7.93)	0.129
Hippuric acid-TMS	Urinary	1.65 (0.60–3.70)	2.62 (0.77–5.62)	1.42 (0.58–3.56)	0.490
Histidine-3TMS	Urinary	3.06 (1.06–6.99)	5.36 (3.09–10.60)	2.19 (0.86–5.51)	0.083
Homocysteine-3TMS (×10^{-2})	Urinary	0.65 (0–2.02)	2.19 (0–3.95)	0.64 (0–1.69)	0.188
Homogentisic acid-3TMS (×10^{-3})	Urinary	0 (0–0)	0 (0–0)	0 (0–0)	0.137
Homovanillic acid-2TMS (×10^{-2})	Urinary	2.09 (1.00–4.50)	3.02 (0.74–5.28)	1.79 (1.04–4.21)	0.635
Hydroquinone-2TMS (×10^{-2})	Urinary	0 (0–1.17)	0 (0–1.63)	0 (0–1.19)	0.898
Hydroxylamine-3TMS	Urinary	0.84 (0.65–1.00)	0.67 (0.50–0.79)	0.90 (0.70–1.05)	**0.007**
Hypotaurine-3TMS (×10^{-2})	Urinary	0 (0–5.66)	0 (0–0.68)	1.94 (0–6.37)	**0.043**
Hypoxanthine-2TMS (×10^{-1})	Urinary	2.16 (1.25–3.43)	2.92 (1.82–3.97)	2.15 (1.14–3.42)	0.366
Indol-3-acetic acid-TMS (×10^{-2})	Urinary	1.75 (0–3.74)	1.50 (0.59–4.03)	1.82 (0–3.87)	0.721

Inositol-6TMS (×10^{-1})	Urinary	2.48 (1.78–3.92)	2.30 (1.77–3.00)	2.70 (1.77–4.10)	0.490
Isobutyrylglycine-TMS	Urinary	0 (0–0)	0 (0–0)	0 (0–0)	0.928
Isocitric acid-4TMS	Urinary	2.99 (2.35–5.69)	4.63 (2.14–5.84)	2.98 (2.40–5.64)	0.890
Isoleucine-2TMS (×10^{-1})	Urinary	3.19 (1.56–4.53)	3.47 (2.91–4.82)	2.83 (1.46–4.34)	0.334
Isovalerylglycine-TMS (×10^{-3})	Urinary	0 (0–0)	0 (0–2.37)	0 (0–0)	0.186
Kynurenic acid-2TMS (×10^{-1})	Urinary	1.17 (0.69–2.00)	1.73 (0.65–3.32)	1.14 (0.73–1.79)	0.290
Kynurenine-2TMS (×10^{-1})	Urinary	0.59 (0.34–1.20)	0.98 (0.36–1.29)	0.52 (0.30–1.10)	0.382
Lactic acid-2TMS	Urinary	0.86 (0.64–1.15)	0.69 (0.55–0.91)	0.97 (0.68–1.17)	**0.045**
Lactose-meto-8TMS (×10^{-2})	Urinary	1.83 (1.05–5.14)	2.44 (0.84–9.05)	1.82 (0.99–4.51)	0.373
Lauric acid-TMS (×10^{-1})	Urinary	0.85 (0.70–1.03)	0.87 (0.68–0.99)	0.84 (0.69–1.10)	0.702
Leucine-2TMS	Urinary	0.97 (0.50–1.48)	1.21 (0.88–1.49)	0.94 (0.49–1.49)	0.417
Linoleic acid-TMS (×10^{-2})	Urinary	1.58 (1.30–2.11)	1.57 (1.48–2.28)	1.59 (1.25–2.10)	0.334
Lysine-4TMS (×10^{-1})	Urinary	3.85 (1.46–6.90)	4.93 (1.98–6.30)	3.69 (1.37–7.88)	0.592
Lyxose-meto-4TMS (×10^{-1})	Urinary	3.54 (2.54–5.31)	4.03 (2.30–6.39)	3.39 (2.49–5.16)	0.399
Maleic acid-2TMS (×10^{-2})	Urinary	2.76 (1.76–5.15)	4.10 (1.91–5.35)	2.70 (1.76–5.06)	0.725
Malic acid-3TMS (×10^{-2})	Urinary	0.43 (0–1.10)	0 (0–1.25)	0.45 (0–1.10)	0.530
Malonic acid-2TMS (×10^{-2})	Urinary	1.63 (1.04–2.29)	1.27 (1.01–2.03)	1.68 (1.12–2.67)	0.263
Mannitol-6TMS	Urinary	1.41 (0.87–2.92)	1.25 (1.09–2.68)	1.43 (0.80–3.05)	0.748
Mannose-meto-5TMS (×10^{-1})	Urinary	0.59 (0.32–0.89)	0.43 (0.20–0.69)	0.64 (0.34–0.91)	0.173
Margaric acid-TMS (×10^{-2})	Urinary	2.67 (2.20–3.40)	2.69 (2.43–3.56)	2.66 (2.15–3.29)	0.530
Mesaconic acid-2TMS (×10^{-1})	Urinary	0.90 (0.57–1.79)	0.85 (0.48–1.15)	1.04 (0.57–1.93)	0.382
meso-Erythritol-4TMS (×10^{-1})	Urinary	2.73 (1.81–4.07)	2.95 (1.83–4.22)	2.68 (1.70–4.08)	0.842
Methionine sulfone-2TMS (×10^{-2})	Urinary	1.29 (0–2.45)	1.51 (0.96–2.67)	1.27 (0–2.47)	0.438
Methionine-2TMS (×10^{-2})	Urinary	0 (0–1.11)	0 (0–1.63)	0 (0–0.87)	0.400
Methylmalonic acid-2TMS (×10^{-2})	Urinary	3.54 (2.75–5.05)	3.38 (2.59–5.55)	3.74 (2.73–5.08)	0.988
Methylsuccinic acid-2TMS (×10^{-2})	Urinary	3.30 (2.42–5.93)	3.09 (2.12–4.06)	3.75 (2.40–6.37)	0.399
Myristic acid-TMS (×10^{-1})	Urinary	1.23 (1.03–1.62)	1.23 (1.08–1.82)	1.23 (1.01–1.63)	0.635
N6-Acetyllysine-2TMS (×10^{-1})	Urinary	1.94 (1.02–3.11)	2.45 (1.86–2.87)	1.51 (0.97–3.37)	0.434
N-Acetylaspartic acid-3TMS (×10^{-1})	Urinary	1.00 (0.54–1.99)	1.88 (0.78–3.03)	0.85 (0.49–1.49)	0.095

N-Acetylglutamine-2TMS (×10^{-2})	Urinary	1.50 (0.54–2.77)	2.59 (0.75–3.71)	1.43 (0.36–2.77)	0.235
N-Acetylmannosamine-meto-4TMS (×10^{-2})	Urinary	3.34 (2.39–4.75)	4.26 (2.24–6.18)	3.15 (2.38–4.47)	0.434
N-Acetylneuraminic acid-6TMS (×10^{-1})	Urinary	1.22 (0.85–1.87)	1.62 (0.77–2.51)	1.14 (0.87–1.68)	0.471
N-Acetylserine-2TMS (×10^{-2})	Urinary	0.83 (0–1.18)	1.15 (0–1.24)	0.59 (0–1.09)	0.355
N-Acetyltyrosine-2TMS	Urinary	0 (0–0)	0 (0–0)	0 (0–0)	0.354
Niacinamide-TMS (×10^{-1})	Urinary	0.77 (0.42–1.18)	0.65 (0.43–1.31)	0.81 (0.42–1.19)	0.635
Nicotinic acid-TMS (×10^{-2})	Urinary	3.42 (1.20–7.61)	0.99 (0.35–1.37)	5.42 (1.97–8.78)	**0.001**
Nonanoic acid-TMS (×10^{-1})	Urinary	3.46 (2.86–4.03)	3.10 (2.53–3.65)	3.53 (2.96–4.06)	0.108
Norepinephrine-5TMS (×10^{-1})	Urinary	1.19 (0.76–1.75)	1.18 (0.58–2.20)	1.19 (0.79–1.77)	0.702
Norvaline-2TMS (×10^{-2})	Urinary	0 (0–1.05)	0 (0–1.01)	0 (0–1.23)	0.722
Octadecanol-TMS (×10^{-1})	Urinary	2.16 (1.94–2.79)	2.24 (2.02–2.98)	2.13 (1.91–2.70)	0.382
Octanoic acid-TMS (×10^{-2})	Urinary	5.80 (4.98–7.31)	5.34 (4.88–7.17)	5.89 (5.14–7.37)	0.453
Oleamide-TMS (×10^{-1})	Urinary	2.50 (1.78–3.78)	2.54 (1.99–4.26)	2.47 (1.67–3.70)	0.571
Oleic acid-TMS (×10^{-1})	Urinary	1.04 (0.73–1.33)	1.22 (1.09–1.35)	0.97 (0.66–1.56)	0.137
O-Phosphoethanolamine-4TMS	Urinary	0 (0–0)	0 (0–0)	0 (0–0)	0.524
Ornithine-4TMS (×10^{-1})	Urinary	3.50 (1.77–6.08)	4.07 (2.01–4.89)	3.50 (1.76–6.32)	0.818
Oxalic acid-2TMS (×10^{-1})	Urinary	3.78 (2.95–4.26)	3.75 (2.65–4.06)	3.78 (3.12–4.50)	0.305
Palmitic acid-TMS	Urinary	3.27 (2.77–3.85)	3.32 (3.03–4.19)	3.26 (2.66–3.80)	0.290
Palmitoleic acid-TMS (×10^{-2})	Urinary	1.06 (0.67–1.93)	1.57 (0.76–2.11)	1.04 (0.64–1.86)	0.489
Pantothenic acid-3TMS (×10^{-2})	Urinary	1.05 (0–2.06)	1.51 (0.60–2.83)	0.95 (0–1.85)	0.330
ParaXanthine-TMS (×10^{-2})	Urinary	3.78 (1.52–8.06)	1.53 (0.33–3.38)	4.67 (2.68–8.45)	**0.016**
Phenylalanine-2TMS	Urinary	1.50 (0.72–1.92)	1.79 (1.28–2.10)	0.96 (0.69–1.88)	0.154
Phenylpyruvic acid-meto-TMS	Urinary	0 (0–0)	0 (0–0)	0 (0–0)	0.602
Phosphoric acid-3TMS	Urinary	137.6 (102.2–171.4)	120.0 (94.3–175.1)	139.7 (101.4–170.6)	0.613
Pimelic acid-2TMS (×10^{-2})	Urinary	0 (0–0.45)	0 (0–0)	0 (0–0.66)	0.259
Proline-2TMS (×10^{-2})	Urinary	4.18 (2.21–6.31)	4.53 (1.85–6.15)	4.02 (2.46–6.44)	0.866
Protocatechuic acid-3TMS (×10^{-3})	Urinary	0 (0–0)	0 (0–2.57)	0 (0–0)	**0.043**
Psicose-meto-5TMS (×10^{-1})	Urinary	1.64 (0.92–5.18)	2.74 (0.49–1.07)	1.41 (1.01–3.65)	0.530
Putrescine-4TMS (×10^{-1})	Urinary	1.35 (0.94–1.92)	1.25 (0.64–1.93)	1.36 (0.97–1.93)	0.613

Pyridoxal-meto-2TMS	Urinary	0 (0–0)	0 (0–0)	0 (0–0)	0.892
Pyrogallol-3TMS ($\times 10^{-1}$)	Urinary	0.75 (0.28–3.24)	0.78 (0.32–11.28)	0.72 (0.27–3.04)	0.530
Pyruvic acid-meto-TMS ($\times 10^{-1}$)	Urinary	3.13 (2.10–3.73)	2.90 (1.62–3.89)	3.19 (2.17–3.70)	0.794
Quinolinic acid-2TMS ($\times 10^{-1}$)	Urinary	3.36 (2.47–5.31)	4.18 (2.30–6.91)	3.32 (2.36–4.24)	0.399
Ribitol-5TMS ($\times 10^{-1}$)	Urinary	0.80 (0.51–1.34)	1.02 (0.66–1.56)	0.66 (0.48–1.29)	0.350
Ribonic acid-5TMS	Urinary	1.45 (0.99–2.01)	1.70 (1.07–2.38)	1.35 (0.98–1.91)	0.203
Ribonolactone-3TMS ($\times 10^{-2}$)	Urinary	5.83 (3.42–8.61)	6.60 (4.05–9.89)	5.81 (3.37–8.49)	0.530
Ribose 5-phosphate-meto-5TMS ($\times 10^{-3}$)	Urinary	0 (0–1.28)	0 (0–8.05)	0 (0–0)	0.176
Ribose-meto-4TMS ($\times 10^{-1}$)	Urinary	0.87 (0.59–1.23)	0.91 (0.55–1.51)	0.83 (0.58–1.12)	0.399
Ribulose-meto-4TMS ($\times 10^{-1}$)	Urinary	1.62 (1.08–2.42)	2.23 (0.97–3.25)	1.54 (1.08–2.27)	0.530
Saccharopine-4TMS	Urinary	0 (0–0)	0 (0–0)	0 (0–0)	0.602
Sebacic acid-2TMS	Urinary	0 (0–0)	0 (0–0)	0 (0–0)	0.354
Serine-3TMS	Urinary	1.67 (1.06–2.78)	1.73 (1.46–3.64)	1.52 (0.91–2.39)	0.290
Sorbitol-6TMS ($\times 10^{-1}$)	Urinary	3.91 (2.82–5.49)	4.53 (3.23–7.23)	3.78 (2.73–4.84)	0.145
Sorbose-meto-5TMS ($\times 10^{-2}$)	Urinary	3.79 (1.90–7.94)	3.87 (1.89–12.82)	3.76 (1.95–7.09)	0.399
Spermidine-5TMS ($\times 10^{-3}$)	Urinary	0 (0–0)	0 (0–8.54)	0 (0–0)	0.688
Spermine-6TMS ($\times 10^{-1}$)	Urinary	1.02 (0.48–1.36)	1.04 (0.64–1.35)	1.01 (0.44–1.36)	0.613
Stearic acid-TMS	Urinary	2.55 (2.34–3.17)	2.45 (2.40–3.20)	2.58 (2.26–3.17)	0.939
Suberic acid-2TMS ($\times 10^{-2}$)	Urinary	0.46 (0–1.76)	0 (0–0.46)	0.88 (0–2.32)	0.101
Succinic acid-2TMS ($\times 10^{-1}$)	Urinary	0.91 (0.68–1.18)	0.95 (0.75–1.28)	0.91 (0.68–1.14)	0.635
Sucrose-8TMS	Urinary	0.18 (0.10–0.38)	1.08 (0.04–1.20)	0.18 (0.11–0.33)	0.250
Tagatose-meto-5TMS ($\times 10^{-1}$)	Urinary	0.38 (0.18–1.19)	0.93 (0.10–1.37)	0.36 (0.18–0.89)	0.334
Tartaric acid-4TMS	Urinary	0.14 (0.07–0.91)	0.12 (0.02–1.01)	0.15 (0.08–0.75)	0.635
Taurine-13C2-3TMS ($\times 10^{-1}$)	Urinary	0.59 (0.28–1.11)	0.49 (0.23–0.88)	0.67 (0.29–1.16)	0.550
Thiodiglycolic acid-2TMS ($\times 10^{-3}$)	Urinary	0 (0–0)	0 (0–6.60)	0 (0–0)	0.052
Threitol-4TMS ($\times 10^{-1}$)	Urinary	1.28 (0.67–1.95)	1.25 (0.53–2.51)	1.31 (0.73– 1.87)	0.988
Threonic acid-4TMS	Urinary	1.54 (0.89–2.28)	2.04 (1.01–2.54)	1.46 (0.88–2.24)	0.350
Threonine-3TMS ($\times 10^{-1}$)	Urinary	3.17 (1.91–4.93)	3.56 (2..69–5.23)	3.14 (1.80–5.04)	0.434
Tiglylglycine-TMS	Urinary	0 (0–0)	0 (0–0)	0 (0–0)	0.892

Trehalose-8TMS (×10⁻²)	Urinary	2.07 (0–7.97)	1.48 (0–16.58)	2.21 (1.05–6.80)	0.963
Triethanolamine-3TMS (×10⁻²)	Urinary	0 (0–1.55)	0 (0–1.51)	0 (0–1.64)	0.956
Tryptophan-3TMS (×10⁻¹)	Urinary	1.36 (0.72–2.11)	1.47 (1.14–1.73)	1.25 (0.65–2.30)	0.890
Tyramine-3TMS (×10⁻¹)	Urinary	2.36 (1.69–3.80)	3.74 (1.94–7.25)	2.34 (1.63–3.61)	0.145
Tyrosine-3TMS	Urinary	3.10 (1.63–5.57)	3.92 (2.29–5.77)	2.97 (1.48–5.51)	0.319
Uracil-2TMS (×10⁻²)	Urinary	0.17 (0–0.85)	0.59 (0–1.71)	0 (0–0.78)	0.232
Urea-2TMS	Urinary	200.3 (151.4–248.7)	172.1 (150.3–237.0)	201.0 (165.3–255.9)	0.702
Ureidopropionic acid-2TMS (×10⁻³)	Urinary	0 (0–8.79)	8.46 (0–9.92)	0 (0–4.93)	0.254
Uric acid-4TMS	Urinary	4.43 (3.07–6.20)	4.48 (3.00–7.53)	4.37 (3.06–5.75)	0.771
Urocanic acid-2TMS (×10⁻¹)	Urinary	1.37 (1.01–2.07)	2.08 (1.03–2.54)	1.25 (0.99–1.69)	0.163
Valine-2TMS	Urinary	1.40 (0.77–1.80)	1.64 (1.11–1.91)	1.27 (0.75–1.80)	0.334
Vanilmandelic acid-3TMS (×10⁻¹)	Urinary	5.01 (2.59–5.90)	5.52 (2.29–8.37)	4.94 (2.76–5.71)	0.471
Xanthine-3TMS (×10⁻¹)	Urinary	0.54 (0.32–0.96)	0.54 (0.37–1.33)	0.55 (0.31–0.92)	0.771
Xanthosine-5TMS (×10⁻²)	Urinary	0.86 (0–1.55)	1.08 (0.27–1.43)	0.82 (0–1.61)	0.518
Xylitol-5TMS (×10⁻¹)	Urinary	1.02 (0.65–1.65)	1.37 (0.64–2.22)	0.97 (0.64–1.54)	0.550
Xylose-meto-4TMS (×10⁻¹)	Urinary	1.17 (0.55–1.79)	1.20 (0.36–1.65)	1.16 (0.59–1.94)	0.510
Xylulose-meto-4TMS (×10⁻¹)	Urinary	1.97 (1.30–3.10)	3.05 (1.05–3.57)	1.87 (1.33–2.80)	0.550

Abbreviations: IQR, Interquartile range; arb.unit, arbitrary unit; AHU, asymptomatic hyperuricemia; Raw P values were calculated from Mann–Whitney U test comparing asymptomatic hyperuricemia group to all gout group (without the Benjamini-Hochberg adjustment).

Table S2. Summary of multivariate and univariate analysis results among asymptomatic hyperuricemia, gout with SUA <9.0 mg/dL, and gout with SUA ≥9.0 mg/dL groups.

Compound name [arb.unit]	Sample	VIP [1]	VIP [2]	Median (IQR: Q1-Q3)			Raw *P* [3]	Raw *P* [4]
				AHU	Gout (SUA <9.0 mg/dL)	Gout (SUA ≥9.0 mg/dL)		
**2-Isopropylmalic acid-3TMS**	**Plasma**			**1**	**1**	**1**	**Reference**	
1,5-Anhydro-glucitol-4TMS (×10^{-2})	Plasma	1.509	1.621	0.86 (0.67–1.48)	0.60 (0–1.09)	0.53 (0–0.89)	0.163	0.073
1,6-Anhydroglucose-3TMS (×10^{-2})	Plasma	0.556	0.115	0.63 (0–1.07)	0.83 (0–1.05)	0.75 (0–0.94)	0.85	0.994
1-Hexadecanol-TMS (×10^{-2})	Plasma	0.349	0.454	2.46 (2.09–2.53)	2.14 (1.63–2.74)	1.87 (1.62–2.52)	0.99	0.413
2-Aminoadipic acid-3TMS (×10^{-3})	Plasma	1.292	0.659	4.88 (0–8.35)	0 (0–5.35)	0 (0–14.40)	0.487	0.94
2-Aminobutyric acid-2TMS (×10^{-2})	Plasma	0.919	0.571	4.02 (3.77–5.79)	4.46 (3.27–6.79)	4.04 (2.98–5.41)	0.942	0.793
2-Aminoethanol-3TMS (×10^{-1})	Plasma	0.511	0.677	3.59 (2.96–4.29)	3.48 (3.04–4.14)	3.44 (2.84–3.99)	0.99	0.793
2-Aminoisobutyric acid-2TMS (×10^{-2})	Plasma	0.682	0.853	6.83 (5.26–8.02)	6.70 (5.62–7.56)	6.30 (5.54–7.41)	0.998	0.968
2-Aminopimelic acid-3TMS (×10^{-1})	Plasma	0.786	1.29	2.14 (1.97–2.48)	2.49 (2.21–)2.69	2.71 (2.33–2.90)	0.396	0.099
2-Deoxy-glucose-4TMS (×10^{-1})	Plasma	1.171	0.705	1.01 (0.83–1.27)	1.13 (0.87–1.78)	1.16 (0.83–1.35)	0.497	0.793
2-Hydroxybutyric acid-2TMS (×10^{-1})	Plasma	0.957	1.445	1.39 (1.09–2.02)	1.61 (1.43–2.05)	2.11 (1.56–2.37)	0.283	0.195
2-Hydroxyglutaric acid-3TMS (×10^{-2})	Plasma	0.743	0.329	1.14 (1.07–1.60)	1.42 (0.92–1.84)	1.41 (0.96–1.64)	0.979	0.984
2-Hydroxyisobutyric acid-2TMS (×10^{-2})	Plasma	0.495	1.025	0.93 (0.80–1.21)	1.05 (0.71–1.97)	1.17 (0.89–1.48)	0.942	0.348
2-Hydroxyisovaleric acid-2TMS (×10^{-2})	Plasma	0.921	0.906	3.55 (3.25–4.31)	3.26 (2.24–7.18)	5.00 (2.92–6.23)	0.962	0.38
2-Ketobutyric acid-meto-TMS (×10^{-2})	Plasma	0.776	1.338	2.14 (1.73–2.64)	2.45 (1.82–2.90)	2.77 (2.31–3.14)	0.532	0.141
2-Ketoglutaric acid-meto-2TMS (×10^{-2})	Plasma	1.876	2.362	5.18 (4.61–5.85)	6.61 (5.66–8.14)	8.11 (7.05–9.14)	**0.027**	**<0.001**
2-Ketoisocaproic acid-meto-TMS (×10^{-1})	Plasma	0.543	0.914	1.22 (1.17–1.60)	1.33 (0.99–1.67)	1.42 (1.17–1.74)	0.998	0.716
2-Keto-isovaleric acid-meto-TMS (×10^{-2})	Plasma	0.957	1.166	2.08 (1.86–2.43)	1.90 (1.47–2.48)	2.39 (2.12–2.87)	0.606	0.264
3-Aminoglutaric acid-2TMS (×10^{-2})	Plasma	1.261	0.496	6.97 (0.34–9.39)	0 (0–7.94)	1.53 (0–9.97)	0.311	0.709
3-Aminoisobutyric acid-3TMS (×10^{-3})	Plasma	1.217	1.481	0 (0–0)	0 (0–7.20)	3.17 (0–15.03)	0.297	0.103
3-Aminopropanoic acid-3TMS (×10^{-2})	Plasma	0.841	0.933	3.96 (2.28–5.78)	3.22 (0–6.56)	4.13 (1.34–10.99)	0.99	0.864
3-Hydroxybutyric acid-2TMS (×10^{-1})	Plasma	1.07	0.872	4.04 (2.67–5.06)	4.04 (3.35–7.70)	3.99 (3.35–7.70)	0.569	0.558
3-Hydroxyglutaric acid-3TMS (×10^{-1})	Plasma	0.568	0.528	8.03 (7.85–8.20)	7.97 (7.80–8.19)	7.96 (7.80–8.19)	0.89	0.83
3-Hydroxyisobutyric acid-2TMS (×10^{-1})	Plasma	1.064	1.043	1.84 (1.25–2.25)	1.68 (1.50–2.33)	2.21 (1.47–2.67)	0.86	0.195

3-Hydroxyisovaleric acid-2TMS (×10^{-2})	Plasma	0.787	1.011	4.79 (3.42–5.29)	4.67 (3.74–5.36)	4.90 (4.34–5.34)	0.793	0.483
3-Hydroxypropionic acid-2TMS (×10^{-2})	Plasma	1.491	0.208	6.45 (5.63–8.04)	7.87 (660–8.33)	6.78 (6.07–7.97)	0.235	0.968
3-Methyl-2-oxovaleric acid-meto-TMS (×10^{-1})	Plasma	0.73	1.236	1.22 (0.96–1.40)	1.16 (0.94–1.64)	1.46 (1.07–1.67)	0.999	0.264
3-Phenyllactic acid-2TMS (×10^{-3})	Plasma	1.003	1.061	0 (0–7.59)	5.25 (0–9.05)	6.06 (0–1.09)	0.557	0.299
4-Aminobutyric acid-3TMS	Plasma	0.685	0.682	0 (0–0)	0 (0–0)	0 (0–0)	0.905	0.878
4-Hydroxyphenyllactic acid-3TMS (×10^{-2})	Plasma	1.042	1.242	5.09 (4.14–7.47)	6.65 (4.60–7.79)	7.25 (5.16–9.54)	0.532	0.195
4-Hydroxyphenylpyruvic acid-meto-2TMS	Plasma	0.704	0.646	0 (0–0)	0 (0–0)	0 (0–0)	0.719	0.706
4-Hydroxyproline-3TMS (×10^{-1})	Plasma	1.266	0.33	3.15 (0.20–4.00)	0.80 (0.07–3.23)	1.06 (0.08–4.95)	0.336	0.923
5-Hydroxymethyl-2-furoic acid-2TMS	Plasma	1.259	0	0 (0–0)	0 (0–0)	0 (0–0)	0.719	0.999
5-Methoxytryptamine-2TMS (×10^{-2})	Plasma	0.339	1.177	1.65 (0.34–2.91)	1.73 (0.47–3.32)	1.32 (0.95–1.69)	0.998	0.793
5-Oxoproline-2TMS	Plasma	1.658	1.541	7.77 (6.71–8.75)	6.86 (5.90–7.71)	6.86 (5.22–7.75)	0.193	0.141
Acetoacetic acid-meto-TMS	Plasma	0.993	0.803	0 (0–0)	0 (0–0)	0 (0–0)	0.483	0.706
Acetylglycine-TMS	Plasma	0.948	0.894	0 (0–0)	0 (0–0)	0 (0–0)	0.483	0.463
Aconitic acid-3TMS (×10^{-3})	Plasma	1.173	1.789	0 (0–0)	0 (0–0)	0 (0–9.43)	0.325	**0.047**
Adipic acid-2TMS (×10^{-3})	Plasma	0.526	0.377	9.25 (2.48–12.48)	8.51 (2.89–14.80)	6.80 (4.28–9.80)	0.917	0.923
Alanine-2TMS	Plasma	0.817	0.488	24.4 (21.1–27.5)	22.6 (20.1–27.4)	25.2 (19.4–28.3)	0.644	0.999
Allose-meto-5TMS (×10^{-2})	Plasma	1.224	1.174	7.50 (5.78–9.90)	9.47 (6.30–11.18)	8.77 (6.61–11.70)	0.497	0.413
Arabitol-5TMS (×10^{-2})	Plasma	0.775	0.785	0 (0–0.88)	0.53 (0–1.08)	0.63 (0–1.37)	0.597	0.473
Arachidonic acid-TMS (×10^{-1})	Plasma	1.384	2.285	0.85 (0.73–1.02)	0.76 (0.52–0.87)	0.56 (0.39–0.76)	0.235	**0.007**
Arginine-3TMS (×10^{-2})	Plasma	0.957	0.327	2.19 (1.12–3.00)	1.83 (0–3.21)	1.90 (0.62–4.15)	0.791	0.948
Asparagine-3TMS (×10^{-2})	Plasma	1.286	0.548	6.38 (0–7.56)	0.67 (0–5.61)	1.65 (0–5.34)	0.5	0.781
Aspartic acid-3TMS (×10^{-2})	Plasma	1.282	0.546	6.46 (0–8.76)	0 (0–7.48)	1.23 (0–8.66)	0.378	0.785
Azelaic acid-2TMS	Plasma	0.696	0.772	0 (0–0)	0 (0–0)	0 (0–0)	0.999	0.866
Benzoic acid-TMS (×10^{-1})	Plasma	1.096	0.62	5.40 (4.68–6.27)	5.91 (4.91–7.50)	5.27 (4.83–6.35)	0.428	0.968
Caproic acid-TMS (×10^{-1})	Plasma	0.51	1.696	1.30 (1.07–1.41)	1.28 (1.07–1.51)	1.09 (0.71–1.21)	0.962	0.087
Cholesterol-TMS (×10^{-2})	Plasma	1.412	0.645	1.30 (0.79–1.51)	0.86 (0–1.14)	0.97 (0.81–1.12)	0.18	0.539
Citramalic acid-3TMS (×10^{-2})	Plasma	0.661	0.363	0.94 (0–1.21)	0.76 (0–0.98)	0.64 (0–1.15)	0.633	0.822
Citric acid-4TMS (×10^{-1})	Plasma	0.529	1.09	3.16 (2.94–3.79)	3.51 (3.17–3.86)	3.94 (3.32–4.39)	0.86	0.264
Citrulline-3TMS	Plasma	0	0.646	0 (0–0)	0 (0–0)	0 (0–0)	0.999	0.706

Creatinine-3TMS (×10^{-3})	Plasma	0.658	0.62	0 (0–6.63)	0 (0–2.46)	0 (0–7.70)	0.784	0.898
Cystamine-nTMS	Plasma	0	0.645	0 (0–0)	0 (0–0)	0 (0–0)	0.999	0.706
Cysteine-3TMS (×10^{-2})	Plasma	1.693	1.054	7.64 (0.31–11.68)	1.70 (0–5.88)	2.14 (0–8.56)	0.147	0.44
Cystine-4TMS (×10^{-2})	Plasma	1.387	1.434	2.58 (1.62–3.01)	3.40 (2.26–4.32)	3.16 (2.58–5.31)	0.157	0.141
Decanoic acid-TMS (×10^{-2})	Plasma	0.616	0.335	6.66 (6.02–7.09)	6.75 (5.76–7.38)	6.24 (5.12–8.14)	0.918	0.793
Docosahexaenoic acid-TMS (×10^{-2})	Plasma	1.612	0.968	0.55 (0–1.45)	1.55 (0.59–2.17)	1.06 (0.19–1.70)	0.134	0.473
Eicosapentaenoic acid-TMS (×10^{-2})	Plasma	1.646	2.4	9.31 (7.38–9.53)	7.51 (5.47–8.92)	5.70 (4.35–7.58)	0.175	**0.008**
Elaidic acid-TMS (×10^{-2})	Plasma	1.186	1.175	4.99 (3.78–6.12)	6.10 (4.94–7.98)	6.81 (5.02–9.88)	0.127	0.077
Fructose-meto-5TMS (×10^{-2})	Plasma	1.016	1.532	2.36 (1.81–5.55)	2.36 (1.36–3.36)	1.48 (1.12–2.35)	0.624	0.087
Fumaric acid-2TMS (×10^{-2})	Plasma	0.355	0.833	1.82 (1.50–2.27)	2.02 (1.87–2.48)	2.00 (1.71–2.49)	0.213	0.38
Galacturonic acid-meto-5TMS (×10^{-2})	Plasma	0.735	0.91	0 (0–0.74)	0 (0–0)	0.25 (0–1.37)	0.86	0.421
Glucaric acid-6TMS (×10^{-2})	Plasma	0.838	1.278	0.91 (0.32–1.13)	1.00 (0.60–1.34)	1.14 (0.91–2.00)	0.567	0.124
Gluconic acid-6TMS (×10^{-2})	Plasma	0.897	0.911	3.58 (2.80–4.27)	3.09 (2.65–3.94)	3.86 (2.87–4.71)	0.682	0.755
Glucosamine-5TMS (×10^{-2})	Plasma	0.347	1.358	1.85 (1.60–2.23)	1.90 (1.50–2.57)	1.66 (0.16–2.08)	0.918	0.446
Glucose-meto-5TMS	Plasma	2.01	1.85	5.88 (4.93–6.75)	7.92 (6.49–9.31)	7.05 (6.24–8.95)	**0.024**	**0.029**
Glucuronic acid-meto-5TMS (×10^{-2})	Plasma	0.549	1.195	1.73 (1.10–2.16)	1.88 (1.25–2.69)	2.25 (1.84–3.07)	0.756	0.157
Glutamic acid-3TMS (×10^{-1})	Plasma	1.392	0.339	4.59 (0.58–7.09)	0.49 (0.09–4.90)	2.82 (0.31–5.84)	0.447	0.113
Glutamine-3TMS (×10^{-1})	Plasma	1.471	0.372	7.33 (1.71–9.59)	3.08 (0.93–7.98)	4.82 (1.42–9.71)	0.337	0.793
Glutaric acid-2TMS (×10^{-2})	Plasma	0.759	0.072	1.65 (1.35–1.76)	1.64 (1.25–2.26)	1.45 (1.01–2.26)	0.81	0.989
Glyceric acid-3TMS (×10^{-1})	Plasma	0.955	0.733	1.28 (1.21–1.68)	1.28 (1.14–1.56)	1.24 (1.10–1.56)	0.962	0.558
Glycerol 3-phosphate-4TMS (×10^{-2})	Plasma	1.154	1.415	0 (0–0.94)	1.01 (0–1.27)	0.90 (0.16–1.35)	0.322	0.177
Glycerol-3TMS (×10^{-1})	Plasma	1.15	0.988	1.06 (0.79–1.23)	1.19 (0.86–1.47)	0.82 (0.76–1.12)	0.497	0.483
Glycine-3TMS	Plasma	1.404	1.086	5.92 (4.09–7.49)	4.86 (3.13–5.77)	4.10 (3.04–5.31)	0.309	0.176
Glycolic acid-2TMS (×10^{-1})	Plasma	0.425	0.457	2.19 (1.91–2.45)	2.12 (1.94–2.48)	2.22 (1.84–2.29)	0.942	0.968
Glyoxylic acid-meto-TMS (×10^{-1})	Plasma	1.162	0.358	5.06 (4.73–5.31)	5.11 (4.66–6.04)	4.75 (4.19–5.40)	0.719	0.755
Histidine-3TMS (×10^{-1})	Plasma	1.067	0.461	1.64 (0.93–2.91)	1.13 (0.26–2.32)	1.50 (0.56–3.94)	0.396	0.83
Homocysteine-3TMS	Plasma	0	0.803	0 (0–0)	0 (0–0)	0 (0–0)	0.999	0.706
Hydroxylamine-3TMS (×10^{-1})	Plasma	1.329	0.681	6.24 (4.48–6.86)	7.61 (5.60–0.967)	6.62 (5.54–8.18)	0.175	0.558
Hypotaurine-3TMS (×10^{-2})	Plasma	0.301	0.641	0.84 (0–1.11)	0.49 (0–1.56)	1.00 (0–1.74)	0.999	0.584

Hypoxanthine-2TMS (×10^{-2})	Plasma	1.003	0.209	0 (0–1.18)	0 (0–0.39)	0 (0–1.09)	0.463	0.979
Indol-3-acetic acid-2TMS (×10^{-2})	Plasma	1.378	0.876	3.89 (3.10–5.10)	3.15 (2.09–3.48)	3.24 (1.53–3.76)	0.175	0.217
Inositol-6TMS (×10^{-1})	Plasma	1.335	0.329	1.31 (0.99–1.75)	1.55 (1.20–2.16)	1.31 (1.07–1.66)	0.235	0.984
Isocitric acid-4TMS (×10^{-2})	Plasma	1.284	1.678	1.89 (1.62–2.13)	2.18 (2.00–2.77)	2.58 (2.18–3.09)	0.127	**0.018**
Isoleucine-2TMS	Plasma	1.127	0.189	3.39 (2.38–3.53)	2.87 (1.36–3.68)	2.82 (1.40–4.04)	0.606	0.637
Kynurenine-3TMS (×10^{-3})	Plasma	0.464	0.765	0 (0–5.36)	0 (0–1.98)	0 (0–8.55)	0.870	0.862
Lactic acid-2TMS	Plasma	1.725	1.519	6.28 (5.63–7.71)	7.86 (6.44–9.34)	8.11 (6.87–13.99)	0.079	**0.045**
Lactose-meto-8TMS	Plasma	0	0.6	0 (0–0)	0 (0–0)	0 (0–0)	0.999	0.706
Lauric acid-TMS (×10^{-1})	Plasma	0.307	1.069	0.99 (0.89–1.03)	0.84 (0.71–1.02)	0.79 (0.64–1.11)	0.287	0.319
Leucine-2TMS	Plasma	1.48	0.393	6.85 (4.59–7.24)	5.66 (2.70–6.28)	5.10 (2.61–6.67)	0.157	0.264
Linoleic acid-TMS (×10^{-1})	Plasma	0.63	0.862	1.44 (1.11–1.68)	1.53 (1.19–1.91)	1.54 (1.34–1.99)	0.86	0.598
Lysine-4TMS	Plasma	1.499	0.256	0.59 (0.51–0.97)	0.53 (0.30–0.77)	0.64 (0.37–1.02)	0.283	0.895
Lyxose-meto-4TMS	Plasma	0.704	0	0 (0–0)	0 (0–0)	0 (0–0)	0.719	0.999
Maleic acid-2TMS	Plasma	1.041	1.142	0 (0–0)	0 (0–0)	0 (0–0)	0.947	0.999
Malic acid-3TMS (×10^{-3})	Plasma	1.506	1.451	4.62 (0–5.33)	0 (0–6.45)	6.46 (4.25–7.93)	0.855	0.061
Mannitol-6TMS	Plasma	1.776	1.294	0.94 (0.78–1.03)	1.14 (0.97–1.35)	1.01 (0.88–1.31)	0.036	0.24
Mannose-meto-5TMS (×10^{-1})	Plasma	1.523	1.18	0.93 (0.85–1.37)	1.33 (1.06–1.59)	1.19 (0.92–1.62)	0.127	0.38
Margaric acid-TMS (×10^{-2})	Plasma	0.728	0.446	4.39 (3.78–5.08)	4.01 (3.36–4.95)	4.04 (3.60–4.86)	0.682	0.716
meso-Erythritol-4TMS (×10^{-3})	Plasma	1.157	0.821	0 (0–0)	0 (0–2.38)	0 (0–0)	0.216	0.463
Methionine-2TMS (×10^{-2})	Plasma	1.127	0.384	2.39 (0–3.73)	0 (0–2.46)	0 (0–2.80)	0.544	0.67
Myristic acid-TMS (×10^{-1})	Plasma	0.973	0.715	1.70 (1.44–1.85)	1.50 (1.20–1.87)	1.48 (1.14–1.75)	0.428	0.348
N6-Acetyllysine-2TMS	Plasma	0.69	0	0 (0–0)	0 (0–0)	0 (0–0)	0.719	0.999
N-Acetylglutamine-3TMS	Plasma	0.805	1.018	0 (0–0)	0 (0–0)	0 (0–0)	0.719	0.304
Niacinamide-TMS	Plasma	0	0.682	0 (0–0)	0 (0–0)	0 (0–0)	0.999	0.706
Nicotinic acid-TMS	Plasma	1.298	0.37	0 (0–0)	0 (0–0)	0 (0–0)	0.309	0.878
Nonanoic acid-TMS (×10^{-1})	Plasma	1.669	3.1	6.84 (6.27–7.29)	4.62 (3.21–7.16)	2.96 (2.56–3.31)	0.193	**<0.001**
Octadecanol-TMS (×10^{-2})	Plasma	1.182	0.986	7.53 (7.22–8.30)	8.37 (6.88–9.44)	8.04 (6.93–9.20)	0.235	0.598
Octanoic acid-TMS (×10^{-2})	Plasma	0.227	1.27	6.29 (5.78–6.65)	6.35 (5.58–6.67)	5.33 (3.86–6.25)	0.999	0.099
Oleamide-TMS (×10^{-1})	Plasma	0.597	1.177	2.74 (2.08–3.88)	2.78 (2.32–5.09)	2.53 (2.02–3.24)	0.86	0.558

Oleic acid-TMS (×10^{-1})	Plasma	0.64	1.336	4.17 (3.06–5.09)	3.92 (3.53–5.55)	5.18 (4.04–6.70)	0.793	0.217
O-Phosphoethanolamine-3TMS (×10^{-3})	Plasma	1.267	0.715	0 (0–0)	0 (0–0)	0 (0–8.82)	0.309	0.513
Ornithine-4TMS	Plasma	1.055	0.188	1.61 (0.75–1.85)	1.33 (0.51–1.70)	1.33 (0.55–2.13)	0.606	0.923
Oxalic acid-2TMS	Plasma	0.574	1.433	3.94 (3.60–4.11)	3.81 (3.33–4.12)	3.14 (2.80–3.79)	0.942	0.087
Palmitic acid-TMS	Plasma	0.839	0.625	5.32 (4.38–5.64)	4.08 (4.00–5.48)	4.64 (3.93–5.70)	0.606	0.867
Palmitoleic acid-TMS (×10^{-2})	Plasma	0.943	1.508	2.55 (2.05–4.64)	3.88 (2.52–5.64)	4.92 (3.37–7.35)	0.462	0.077
ParaXanthine-TMS	Plasma	0	0.821	0 (0–0)	0 (0–0)	0 (0–0)	0.999	0.463
Phenylacetic acid-TMS	Plasma	0.789	1.106	0 (0–0)	0 (0–0)	0 (0–0)	0.719	0.304
Phenylalanine-2TMS	Plasma	1.125	0.516	1.75 (0.09–2.57)	0.11 (0.06–2.25)	0.47 (0.07–2.32)	0.428	0.793
Phosphoric acid-3TMS	Plasma	1.712	1.774	23.9 (20.6–27.6)	20.3 (19.7–24.4)	19.9 (17.2–22.0)	0.141	0.039
Proline-2TMS	Plasma	1.074	0.455	2.64 (1.92–4.42)	2.07 (1.22–3.34)	1.73 (1.14–5.40)	0.235	0.637
Psicose-meto-5TMS (×10^{-2})	Plasma	1.105	1.614	3.16 (1.79–5.00)	2.09 (0.36–4.23)	1.72 (1.25–2.36)	0.365	0.087
Putrescine-4TMS (×10^{-2})	Plasma	0.994	0.419	0 (0–1.71)	1.48 (0–1.72)	0 (0–1.44)	0.842	0.979
Pyrogallol-3TMS (×10^{-3})	Plasma	1.69	1.507	0 (0–5.30)	0 (0–0)	0 (0–0)	0.093	0.106
Pyruvic acid-meto-TMS	Plasma	1.036	1.919	0.60 (0.54–0.67)	0.66 (0.46–0.80)	0.79 (0.61–1.03)	0.462	**0.021**
Ribitol-5TMS (×10^{-3})	Plasma	0.817	0.677	0 (0–6.82)	4.87 (0–8.59)	6.31 (0–9.96)	0.325	0.33
Ribonic acid-5TMS (×10^{-2})	Plasma	0.671	0.652	0 (0–2.04)	2.06 (0–2.75)	2.23 (0.34–2.85)	0.446	0.25
Serine-3TMS (×10^{-1})	Plasma	1.531	0.836	9.76 (4.59–11.24)	4.24 (1.81–9.10)	4.11 (1.65–9.03)	0.175	0.24
Sorbitol-6TMS (×10^{-1})	Plasma	0.936	1.938	1.95 (0.81–2.40)	1.78 (1.08–2.09)	1.13 (0.79–1.37)	0.942	0.195
Sorbose-meto-5TMS (×10^{-2})	Plasma	0.976	1.771	2.14 (0.61–4.31)	1.33 (0–2.15)	0 (0–1.69)	0.377	**0.025**
Stearic acid-TMS	Plasma	1.074	0.173	3.70 (3.34–4.06)	3.07 (2.86–3.87)	3.30 (2.86–3.70)	0.283	0.319
Succinic acid-2TMS (×10^{-1})	Plasma	0.554	0.963	0.86 (0.83–0.97)	0.94 (0.81–1.07)	0.85 (0.70–0.92)	0.827	0.38
Sucrose-8TMS (×10^{-2})	Plasma	1.934	1.319	1.69 (0–6.92)	0 (0–0.32)	0 (0–1.14)	**0.044**	0.194
Tagatose-meto-5TMS (×10^{-2})	Plasma	1.004	1.557	2.12 (0–5.66)	1.39 (0.35–2.26)	0 (0–1.45)	0.563	0.173
Tartaric acid-4TMS (×10^{-3})	Plasma	1.141	0.814	0 (0–0)	0 (0–2.76)	0 (0–0)	0.216	0.463
Taurine-3TMS (×10^{-2})	Plasma	1.232	0.819	1.16 (0.50–2.54)	0.98 (0–1.79)	1.88 (1.04–3.42)	0.671	0.412
Threitol-4TMS	Plasma	0.789	0.646	0 (0–0)	0 (0–0)	0 (0–0)	0.719	0.706
Threonic acid-4TMS (×10^{-2})	Plasma	0.744	0.45	3.63 (2.60–4.46)	4.06 (3.06–4.76)	2.99 (2.31–4.30)	0.682	0.755
Threonine-3TMS (×10^{-1})	Plasma	1.05	0.576	6.18 (3.43–6.73)	3.42 (1.40–6.35)	2.99 (1.34–7.37)	0.366	0.38

Triethanolamine-3TMS (×10-2)	Plasma	0.916	0.879	1.58 (1.10–2.90)	1.66 (1.46–2.24)	1.51 (0.96–1.89)	0.942	0.598
Tryptophan-3TMS	Plasma	1.3	1.564	1.58 (1.19–2.20)	1.26 (0.85–1.74)	1.10 (0.81–1.57)	0.235	0.087
Tyramine-3TMS	Plasma	1.522	0.234	1.27 (0.97–1.91)	1.10 (0.62–1.47)	1.29 (0.72–1.95)	0.337	0.923
Tyrosine-3TMS	Plasma	1.044	0.274	3.44 (2.98–4.46)	2.74 (2.22–4.33)	3.25 (2.14–5.08)	0.682	0.83
Urea-2TMS	Plasma	0.63	0.246	10.81 (8.43–11.52)	8.95 (8.28–12.27)	8.90 (6.87–13.30)	0.756	0.677
Uric acid-4TMS	Plasma	0.888	2.221	1.46 (1.39–1.78)	1.53 (1.38–1.56)	1.92 (1.81–2.32)	0.942	**0.009**
Valine-2TMS	Plasma	1.316	0.688	15.5 (13.6–16.6)	13.8 (10.9–15.7)	12.1 (9.9–17.4)	0.309	0.348
Xanthine-3TMS	Plasma	1.047	0	0 (0–0)	0 (0–0)	0 (0–0)	0.719	0.999
Xylitol-5TMS	Plasma	1.047	0	0 (0–0)	0 (0–0)	0 (0–0)	0.719	0.999
Xylose-meto-4TMS	Plasma	0.898	0	0 (0–0)	0 (0–0)	0 (0–0)	0.483	0.999
**2-Isopropylmalic acid-3TMS**	**Urinary**			**1**	**1**	**1**	**Reference**	
1,6-Anhydroglucose-3TMS (×10-1)	Urinary	0.476	0.681	2.17 (0.59–5.83)	3.46 (2.14–4.18)	2.27 (1.05–3.07)	0.681	0.829
1-Hexadecanol-TMS (×10-1)	Urinary	0.502	1.496	1.93 (1.56–2.27)	1.89 (1.61–2.39)	1.56 (1.33–1.87)	0.999	0.111
2-Aminobutyric acid-2TMS (×10-2)	Urinary	1.073	0.621	1.28 (1.13–1.59)	1.32 (0.69–2.79)	0.95 (0.46–1.60)	0.999	0.157
2-Aminoethanol-3TMS	Urinary	0.658	0.973	14.79 (8.73–20.02)	11.55 (9.36–17.01)	9.59 (5.35–18.19)	0.89	0.598
2-Aminopimelic acid-3TMS	Urinary	0.67	0.934	10.16 (5.04–15.38)	10.02 (7.29–14.78)	7.78 (5.53–9.33)	0.99	0.677
2-Hydroxybutyric acid-2TMS	Urinary	1.047	0.721	0 (0–0)	0 (0–0)	0 (0–0)	0.719	0.463
2-Hydroxyglutaric acid-3TMS (×10-1)	Urinary	0.531	0.65	5.90 (1.94–8.78)	4.30 (3.31–9.28)	4.57 (2.87–7.35)	0.998	0.793
2-Hydroxyhippuric acid-2TMS (×10-2)	Urinary	0.344	0.75	4.72 (1.98–11.03)	3.42 (1.60–6.32)	2.68 (1.29–25.35)	0.625	0.676
2-Hydroxyisobutyric acid-2TMS (×10-1)	Urinary	0.98	0.574	1.28 (0.67–2.07)	1.08 (0.86 -1.62)	1.41 (0.93–2.27)	0.942	0.793
2-Hydroxyisovaleric acid-2TMS	Urinary	1.109	0.955	0 (0–0)	0 (0–0)	0 (0–0)	0.483	0.304
2-Ketoadipic acid-oxime-3TMS	Urinary	1.047	0.598	0 (0–0)	0 (0–0)	0 (0–0)	0.719	0.706
2-Ketobutyric acid-meto-TMS (×10-2)	Urinary	1.321	1.059	1.38 (1.13–1.79)	1.64 (1.27–1.96)	1.60 (1.30–1.88)	0.309	0.413
2-Ketoglutaric acid-meto-2TMS	Urinary	0.675	0.627	1.17 (0.29–1.54)	0.97 (0.58–1.30)	0.77 (0.56–1.02)	0.918	0.923
2-Ketoisocaproic acid-meto-TMS (×10-3)	Urinary	0.438	0.607	6.61 (0–9.61)	0 (0–12.93)	0 (0–8.77)	0.989	0.67
2-Keto-isovaleric acid-meto-TMS	Urinary	1.065	0.803	0 (0–0)	0 (0–0)	0 (0–0)	0.483	0.706
2-Methyl-3-hydroxybutyric acid-2TMS (×10-3)	Urinary	0.728	1.103	0 (0–0)	0 (0–0)	0 (0–7.75)	0.947	0.295
2-Phosphoglyceric acid -4TMS (×10-3)	Urinary	1.568	0.981	0 (0–6.60)	0 (0–0)	0 (0–0)	0.254	0.419
3-Aminoglutaric acid-2TMS (×10-2)	Urinary	1.07	1.89	7.64 (5.35–10.38)	5.41 (4.08–10.96)	3.33 (2.45–6.01)	0.682	**0.039**
3-Aminoisobutyric acid-3TMS (×10-1)	Urinary	0.818	0.552	1.31 (0.79–15.74)	5.20 (0.86–16.84)	1.18 (0.49–6.01)	0.756	0.716

3-Aminopropanoic acid-3TMS (×10^{-1})	Urinary	1.052	0.977	0.80 (0.32–1.18)	1.17 (0.36–3.20)	0.82 (0.54–2.34)	0.606	0.793
3-Hydroxy-3-methylglutaric acid-3TMS (×10^{-2})	Urinary	0.713	0.826	6.68 (2.04–7.69)	4.60 (3.56–7.49)	5.29 (4.66–7.20)	0.998	0.948
3-Hydroxyanthranilic acid-3TMS (×10^{-3})	Urinary	0.795	0.917	0 (0–2.72)	0 (0–4.98)	0 (0–1.36)	0.948	0.443
3-Hydroxybutyric acid-2TMS (×10^{-1})	Urinary	1.254	1.403	1.75 (1.35–2.15)	1.98 (1.61–2.36)	2.06 (1.64–2.57)	0.309	0.141
3-Hydroxyglutaric acid-3TMS (×10^{-1})	Urinary	0.605	1.347	8.12 (7.93–8.23)	8.22 (7.55–8.50)	7.68 (7.43–8.01)	0.999	**0.045**
3-Hydroxyisobutyric acid-2TMS (×10^{-1})	Urinary	0.687	0.961	1.79 (1.01–2.05)	1.38 (1.21– 1.89)	1.76 (1.26 –2.99)	0.99	0.637
3-Hydroxyisovaleric acid-2TMS (×10^{-1})	Urinary	0.834	0.94	4.64 (2.80–5.33)	4.02 (3.05–6.59)	4.14 (3.25–7.86)	0.942	0.83
3-Hydroxyphenylacetic acid-2TMS (×10^{-2})	Urinary	0.961	0.168	0 (0–1.15)	0 (0–0.71)	0 (0–1.65)	0.72	0.979
3-Hydroxypropionic acid-2TMS (×10^{-1})	Urinary	0.443	0.58	1.31 (0.77–2.25)	1.10 (0.88–1.97)	1.30 (0.85–1.81)	0.99	0.968
3-Methoxy-4-hydroxybenzoic acid-2TMS (×10^{-2})	Urinary	1.731	1.065	0.77 (0–1.08)	1.57 (0.69–2.37)	0.94 (0–1.84)	**0.049**	0.665
3-Methyl-2-oxovaleric acid-meto-TMS (×10^{-2})	Urinary	0.699	0.92	0.88 (0.64–1.70)	1.47 (0–2.10)	1.53 (0.86–2.11)	0.874	0.637
3-Methyladipic acid-2TMS (×10^{-2})	Urinary	0.86	0.989	0 (0–3.04)	2.82 (0.86–5.13)	3.00 (0–8.24)	0.245	0.503
3-Methylglutaric acid-2TMS (×10^{-2})	Urinary	0.5	0.661	1.48 (1.15–2.23)	1.55 (0.47–2.89)	1.82 (0.29–3.07)	0.99	0.947
3-Phenyllactic acid-2TMS (×10^{-2})	Urinary	1.038	0.989	0 (0–0.56)	0 (0–2.10)	1.04 (0–1.51)	0.627	0.184
3-Phosphoglyceric acid-4TMS (×10^{-3})	Urinary	1.476	0.906	7.35 (0–18.50)	0 (0–9.49)	3.24 (0–22.85)	0.407	0.731
3-Sulfinoalanine-3TMS (×10^{-3})	Urinary	0.971	0.653	0 (0–4.51)	0 (0–3.98)	0 (0–0)	0.728	0.432
4-Aminobutyric acid-3TMS (×10^{-2})	Urinary	1.198	1.854	7.35 (3.82–9.97)	4.27 (0.81–6.99)	1.80 (0–5.99)	0.307	0.051
4-Hydroxybenzoic acid-2TMS (×10^{-2})	Urinary	0.822	0.523	2.70 (1.72–3.99)	3.29 (2.09–4.43)	2.95 (1.25–3.52)	0.682	0.83
4-Hydroxyphenylacetic acid-2TMS (×10^{-1})	Urinary	0.99	0.382	0.93 (0.29–1.51)	1.10 (0.51–2.92)	0.61 (0.34–1.37)	0.497	0.978
4-Hydroxyphenyllactic acid-3TMS (×10^{-1})	Urinary	0.554	0.636	1.32 (0.41–1.78)	1.23 (0.87–1.86)	1.91 (0.68–2.79)	0.942	0.558
4-Hydroxyproline-3TMS (×10^{-2})	Urinary	0.772	0.514	0 (0–9.19)	0 (0–5.17)	0 (0–5.39)	0.988	0.992
5-Aminolevulinic acid-meto-3TMS (×10^{-2})	Urinary	0.783	1.713	1.72 (1.14–2.39)	1.39 (1.03–1.92)	0.86 (0–1.29)	0.701	**0.044**
5-Aminovaleric acid-3TMS (×10^{-1})	Urinary	0.441	1.095	0.76 (0.52–1.33)	0.87 (0.66–1.48)	0.59 (0.35–1.02)	0.918	0.38
5-Hydroxymethyl-2-furoic acid-2TMS (×10^{-1})	Urinary	1.157	0.402	1.84 (0.42–2.66)	1.68 (0.56–3.54)	0.89 (0.71–1.66)	0.89	0.637
5-Oxoproline-2TMS	Urinary	0.813	0.534	10.09 (6.18–11.52)	8.51 (7.41–12.13)	8.44 (7.47–10.96)	0.918	0.948
7-Methylguanine-2TMS (×10^{-1})	Urinary	1.088	0.893	4.00 (2.32–8.43)	3.71 (2.37–5.69)	2.44 (1.86–4.27)	0.569	0.319
Acetoacetic acid-meto-TMS	Urinary	0	0.803	0 (0–0)	0 (0–0)	0 (0–0)	0.999	0.706
Acetylglycine-TMS	Urinary	1.011	0.598	0 (0–0)	0 (0–0)	0 (0–0)	0.483	0.706
Aconitic acid-3TMS	Urinary	0.62	1.08	2.63 (0.89–2.92)	1.97 (1.06–3.05)	1.20 (0.91–2.04)	0.99	0.319

Adenine-2TMS (×10^{-1})	Urinary	1.266	1.353	1.72 (1.17–4.07)	1.63 (1.31–2.42)	1.54 (1.04–2.04)	0.86	0.413
Adenosine-4TMS (×10^{-1})	Urinary	1.109	1.77	1.31 (0.78–2.39)	1.00 (0.73–1.64)	0.71 (0.50–0.97)	0.569	0.077
Adipic acid-2TMS (×10^{-2})	Urinary	0.466	1.104	1.32 (0.78–1.97)	1.60 (1.05–2.68)	2.44 (1.18–4.75)	0.606	0.157
Alanine-2TMS	Urinary	0.942	0.946	12.72 (8.70–13.82)	12.08 (6.50–21.25)	9.17 (5.82–12.13)	0.99	0.38
Allantoin-3TMS (×10^{-2})	Urinary	0.753	0.786	4.74 (0.84–1.11)	4.35 (2.94–7.77)	3.39 (0.22–9.52)	0.962	0.894
Allose-meto-5TMS (×10^{-2})	Urinary	1.108	1.369	7.43 (4.22–24.13)	6.72 (5.22–10.85)	6.60 (3.89–8.29)	0.719	0.483
Arabinose-meto-4TMS (×10^{-1})	Urinary	0.483	1.037	3.98 (2.15–6.26)	4.12 (2.37–5.41)	2.83 (2.35–3.44)	0.999	0.264
Arabitol-5TMS (×10^{-1})	Urinary	0.832	0.603	8.37 (4.25–8.74)	6.59 (4.37–11.06)	5.70 (4.07–10.02)	0.998	0.968
Arachidonic acid-TMS	Urinary	0	0.671	0 (0–0)	0 (0–0)	0 (0–0)	0.999	0.706
Arginine-3TMS (×10^{-1})	Urinary	0.817	1.223	1.51 (1.14–2.33)	1.15 (0.60–2.11)	0.54 (0.17–1.43)	0.644	0.111
Ascorbic acid-4TMS	Urinary	0.979	1.068	0 (0–0)	0 (0–0)	0 (0–0)	0.808	0.331
Asparagine-3TMS (×10^{-1})	Urinary	0.815	0.786	1.14 (0–3.21)	1.38 (0.83–3.82)	1.21 (0.74–2.87)	0.363	0.696
Aspartic acid-3TMS (×10^{-2})	Urinary	1.024	1.876	6.32 (4.82–9.39)	5.98 (2.58–10.06)	3.05 (1.59–5.05)	0.793	**0.045**
Azelaic acid-2TMS (×10^{-2})	Urinary	1.078	0.823	0 (0–0.36)	1.14 (0–4.30)	1.52 (0–2.72)	0.067	0.106
Benzoic acid-TMS (×10^{-1})	Urinary	0.259	0.492	4.45 (3.68–4.80)	4.17 (3.19–4.89)	4.88 (3.62–5.27)	0.918	0.38
Caproic acid-TMS (×10^{-1})	Urinary	0.306	0.393	1.13 (0.93–1.33)	1.17 (0.93–1.30)	1.07 (0.99–1.21)	0.99	0.984
Catechol-2TMS (×10^{-2})	Urinary	0.473	0.72	3.18 (2.16–8.76)	2.95 (1.53–6.34)	2.03 (0.91–5.17)	0.86	0.539
Cholesterol-TMS	Urinary	0.69	0	0 (0–0)	0 (0–0)	0 (0–0)	0.999	0.719
Citramalic acid-3TMS (×10^{-1})	Urinary	1.338	0.617	3.88 (2.20–9.98)	3.95 (2.37–4.73)	3.92 (3.26–6.29)	0.682	0.999
Citric acid-4TMS	Urinary	0.652	0.929	14.94 (7.67–23.22)	15.10 (10.59–21.93)	12.17 (8.30–13.93)	0.979	0.637
Creatinine-3TMS	Urinary	1.649	1.797	1.02 (0.53–2.00)	0.64 (0.40–1.11)	0.57 (0.37–0.80)	0.175	0.125
Cystathionine-4TMS (×10^{-2})	Urinary	1.224	0.404	0 (0–0.94)	1.07 (0–2.15)	0 (0–1.55)	0.154	0.957
Cysteine-3TMS (×10^{-1})	Urinary	0.7	0.827	2.75 (1.64–4.74)	2.42 (1.56–3.93)	1.67 (0.83–2.56)	0.793	0.195
Cystine-4TMS (×10^{-2})	Urinary	0.498	0.694	3.64 (3.04–4.43)	4.62 (1.39–8.38)	1.96 (0–4.66)	0.86	0.097
Cytosine-2TMS (×10^{-1})	Urinary	0.688	1.323	1.79 (0.66–2.67)	1.61 (1.07–2.26)	1.24 (0.92–1.61)	0.979	0.447
Decanoic acid-TMS (×10^{-2})	Urinary	0.646	0.893	5.98 (4.55–7.39)	5.47 (4.59–6.66)	5.17 (4.63–6.41)	0.827	0.637
Dihydroxyacetone phosphate-meto-3TMS (×10^{-1})	Urinary	0.678	0.632	1.52 (0.63–1.94)	1.14 (0.51–1.88)	0.97 (0.60–1.46)	0.89	0.38
Docosahexaenoic acid-TMS (×10^{-2})	Urinary	0.737	0.303	0 (0–1.40)	0 (0–1.24)	0 (0–1.10)	0.994	0.934
Elaidic acid-TMS (×10^{-1})	Urinary	0.781	0.353	0.94 (0.87–1.20)	0.83 (0.54–1.76)	0.90 (0.60–1.49)	0.644	0.716

Ethylmalonic acid-2TMS ($\times 10^{-1}$)	Urinary	0.551	0.661	0.96 (0.46–1.11)	0.82 (0.52–1.19)	0.81 (0.51–1.29)	0.962	0.994
Fructose-meto-5TMS ($\times 10^{-2}$)	Urinary	0.673	1.531	7.50 (1.47–15.30)	4.01 (2.05–8.43)	3.54 (1.69–6.46)	0.644	0.348
Fucose-meto-4TMS ($\times 10^{-2}$)	Urinary	1.074	0.613	0 (0–1.89)	1.44 (0–1.68)	0 (0–1.09)	0.5	0.882
Fumaric acid-2TMS ($\times 10^{-2}$)	Urinary	1.443	0.966	5.23 (3.05–5.98)	6.81 (4.52–8.85)	6.48 (4.07–7.44)	0.235	0.319
Galactitol-6TMS ($\times 10^{-1}$)	Urinary	1.487	1.37	1.64 (1.15–4.71)	1.43 (0.75–2.51)	1.13 (0.77–2.05)	0.532	0.319
Galacturonic acid-meto-5TMS	Urinary	0.493	0.625	1.56 (0.74–2.55)	1.24 (1.05–2.06)	1.07 (0.72–1.59)	0.979	0.447
Glucaric acid-6TMS	Urinary	0.845	0.582	1.45 (0.86–2.13)	1.16 (0.90–1.58)	1.04 (0.77–1.34)	0.827	0.413
Gluconic acid-6TMS	Urinary	1.3	1.557	5.08 (1.93–5.51)	2.82 (1.75–4.00)	2.68 (2.00–3.66)	0.337	0.195
Glucosamine-5TMS ($\times 10^{-1}$)	Urinary	0.564	0.878	5.78 (3.13–6.95)	3.59 (3.19–7.12)	2.67 (1.38–5.82)	0.99	0.125
Glucose 6-phosphate-meto-6TMS	Urinary	0	0.803	0 (0–0)	0 (0–0)	0 (0–0)	0.999	0.706
Glucose-meto-5TMS ($\times 10^{-1}$)	Urinary	0.681	0.491	5.20 (2.60–8.50)	4.83 (3.32–9.02)	3.83 (2.97–6.40)	0.942	0.895
Glucuronic acid-meto-5TMS	Urinary	0.696	0.6	3.83 (1.57–5.51)	3.35 (2.27–5.06)	3.12 (1.77–3.92)	0.99	0.793
Glutamic acid-3TMS ($\times 10^{-1}$)	Urinary	0.92	1.273	2.72 (1.44–3.29)	1.88 (1.61–2.70)	1.50 (1.17–2.55)	0.719	0.195
Glutamine-3TMS ($\times 10^{-1}$)	Urinary	0.765	0.948	3.65 (2.60–6.14)	2.52 (1.82–5.40)	1.46 (0.85–4.98)	0.756	0.176
Glyceric acid-3TMS ($\times 10^{-1}$)	Urinary	0.809	0.84	1.50 (0.82–1.89)	1.93 (1.11–2.17)	1.84 (0.93–3.00)	0.396	0.52
Glycerol 3-phosphate-4TMS ($\times 10^{-1}$)	Urinary	0.699	0.625	3.70 (1.68–4.43)	2.51 (1.28–4.74)	2.46 (1.48–3.44)	0.918	0.348
Glycine-3TMS	Urinary	1.047	1.229	17.50 (12.37–28.87)	11.84 (8.86–25.98)	8.68 (6.68–18.71)	0.337	0.217
Glycolic acid-2TMS	Urinary	0.979	0.444	1.48 (0.87–2.38)	1.17 (0.83–1.61)	1.43 (1.01–1.87)	0.428	0.994
Glycyl-Glycine-4TMS	Urinary	1.301	1.111	0 (0–0)	0 (0–0)	0 (0–0)	0.309	0.331
Guanine-3TMS ($\times 10^{-2}$)	Urinary	0.791	1.745	7.87 (4.98–11.56)	6.67 (3.47–11.63)	4.37 (1.68–7.72)	0.644	0.087
Hippuric acid-TMS	Urinary	1.333	0.07	2.62 (0.77–5.62)	1.53 (0.73–3.83)	1.19 (0.35–3.44)	0.756	0.755
Histidine-3TMS	Urinary	1.038	1.01	5.36 (3.09–10.60)	2.36 (1.49–6.11)	1.39 (0.54–5.01)	0.258	0.157
Homocysteine-3TMS ($\times 10^{-2}$)	Urinary	1.313	0.584	2.19 (0–3.95)	1.17 (0–1.90)	0.27 (0–1.03)	0.519	0.263
Homogentisic acid-3TMS ($\times 10^{-3}$)	Urinary	1.254	1.05	0 (0–0)	0 (0–2.12)	0 (0–0)	0.216	0.304
Homovanillic acid-2TMS ($\times 10^{-2}$)	Urinary	0.72	0.253	3.02 (0.74–5.28)	2.40 (1.10–4.22)	1.62 (0.80–4.26)	0.99	0.677
Hydroquinone-2TMS ($\times 10^{-2}$)	Urinary	0.432	0.206	0 (0–1.63)	0 (0–1.19)	0 (0–1.42)	0.997	0.991
Hydroxylamine-3TMS	Urinary	1.83	1.883	0.67 (0.50–0.79)	0.89 (0.63–1.07)	0.92 (0.75–1.04)	0.101	**0.006**
Hypotaurine-3TMS ($\times 10^{-2}$)	Urinary	1.652	1.534	0 (0–0.68)	0 (0–6.53)	2.31 (0–6.42)	0.17	0.069
Hypoxanthine-2TMS ($\times 10^{-1}$)	Urinary	0.618	0.93	2.92 (1.82–3.97)	1.32 (2.17–4.23)	1.68 (0.83–3.16)	0.979	0.24

Indol-3-acetic acid-TMS (×10^{-2})	Urinary	0.822	0.837	1.50 (0.59–4.03)	1.75 (0–2.89)	2.75 (0–4.98)	0.999	0.751
Inositol-6TMS (×10^{-1})	Urinary	1.217	0.852	2.30 (1.77–3.00)	3.21 (1.97–4.28)	2.41 (1.55–3.87)	0.396	0.999
Isobutyrylglycine-TMS	Urinary	0.618	0.354	0 (0–0)	0 (0–0)	0 (0–0)	0.992	0.928
Isocitric acid-4TMS	Urinary	0.558	0.556	4.63 (2.14–5.84)	3.16 (2.43–5.84)	2.81 (1.94–5.18)	0.979	0.864
Isoleucine-2TMS (×10^{-1})	Urinary	0.78	0.977	3.47 (2.91–4.82)	3.66 (1.69–4.78)	1.83 (1.16–4.03)	0.942	0.24
Isovalerylglycine-TMS (×10^{-3})	Urinary	1.017	0.546	0 (0–2.37)	0 (0–0)	0 (0–0)	0.434	0.476
Kynurenic acid-2TMS (×10^{-1})	Urinary	1.396	1.255	1.73 (0.65–3.32)	1.33 (0.71–1.95)	1.03 (0.71–1.61)	0.569	0.483
Kynurenine-2TMS (×10^{-1})	Urinary	0.723	0.805	0.98 (0.36–1.29)	0.71 (0.47–1.25)	0.39 (0.20–0.70)	0.999	0.176
Lactic acid-2TMS	Urinary	1.177	1.584	0.69 (0.55–0.91)	0.92 (0.63–1.11)	1.00 (0.69–1.20)	0.213	0.067
Lactose-meto-8TMS (×10^{-2})	Urinary	1.283	1.223	2.44 (0.84–9.05)	1.85 (1.32–5.05)	1.34 (0.63–4.37)	0.827	0.429
Lauric acid-TMS (×10^{-1})	Urinary	0.967	0.626	0.87 (0.68–0.99)	0.79 (0.69–1.24)	0.88 (0.68–0.99)	0.89	0.945
Leucine-2TMS	Urinary	0.756	0.885	1.21 (0.88–1.49)	0.98 (0.52–1.50)	0.66 (0.44–1.46)	0.89	0.447
Linoleic acid-TMS (×10^{-2})	Urinary	0.592	0.438	1.57 (1.48–2.28)	1.68 (1.25–2.10)	1.55 (1.21–2.04)	0.606	0.558
Lysine-4TMS (×10^{-1})	Urinary	0.551	0.568	4.93 (1.98–6.30)	4.63 (2.66–8.84)	1.80 (1.16–4.84)	0.942	0.319
Lyxose-meto-4TMS (×10^{-1})	Urinary	0.483	1.062	4.03 (2.30–6.39)	4.33 (2.50–5.43)	2.94 (2.45–3.58)	0.99	0.264
Maleic acid-2TMS (×10^{-2})	Urinary	0.964	1.25	4.10 (1.91–5.35)	3.21 (1.76–5.39)	2.58 (1.56–4.55)	0.999	0.716
Malic acid-3TMS (×10^{-2})	Urinary	0.911	0.891	0 (0–1.25)	0.67 (0–1.21)	0.43 (0–1.01)	0.715	0.866
Malonic acid-2TMS (×10^{-2})	Urinary	1.701	0.477	1.27 (1.01–2.03)	2.19 (1.68–2.94)	1.30 (0.85–1.59)	0.062	0.984
Mannitol-6TMS	Urinary	0.927	0.724	1.25 (1.09–2.68)	1.93 (0.87–3.10)	1.30 (0.70–2.84)	0.998	0.716
Mannose-meto-5TMS (×10^{-1})	Urinary	0.72	0.566	0.43 (0.20–0.69)	0.68 (0.48–1.22)	0.59 (0.28–0.88)	0.175	0.637
Margaric acid-TMS (×10^{-2})	Urinary	0.705	0.419	2.69 (2.43–3.56)	2.67 (2.07–3.29)	2.64 (2.17–3.43)	0.827	0.755
Mesaconic acid-2TMS (×10^{-1})	Urinary	1.425	0.763	0.85 (0.48–1.15)	1.29 (0.55–1.93)	0.78 (0.60–1.84)	0.366	0.923
meso-Erythritol-4TMS (×10^{-1})	Urinary	0.955	0.576	2.95 (1.83–4.22)	3.81 (2.04–4.37)	2.05 (1.50–3.12)	0.827	0.558
Methionine sulfone-2TMS (×10^{-2})	Urinary	0.863	1.097	1.51 (0.96–2.67)	1.53 (0.62–2.54)	0.80 (0–2.18)	0.99	0.197
Methionine-2TMS (×10^{-2})	Urinary	0.876	1.042	0 (0–1.63)	0 (0–1.13)	0 (0–0.76)	0.792	0.528
Methylmalonic acid-2TMS (×10^{-2})	Urinary	0.551	0.987	3.38 (2.59–5.55)	4.03 (2.83–5.84)	3.49 (2.67–4.19)	0.89	0.864
Methylsuccinic acid-2TMS (×10^{-2})	Urinary	0.272	0.835	3.09 (2.12–4.06)	3.39 (2.46–5.23)	5.46 (2.27–7.31)	0.89	0.413
Myristic acid-TMS (×10^{-1})	Urinary	0.569	0.284	1.23 (1.08–1.82)	1.18 (0.89–1.63)	1.26 (1.07–1.65)	0.606	0.999
N6-Acetyllysine-2TMS (×10^{-1})	Urinary	0.567	0.832	2.45 (1.86–2.87)	2.05 (1.12–3.37)	1.13 (0.42–3.52)	0.99	0.319

N-Acetylaspartic acid-3TMS (×10^{-1})	Urinary	1.21	1.46	1.88 (0.78–3.03)	1.18 (0.67–1.93)	0.70 (0.32–1.12)	0.497	0.077
N-Acetylglutamine-2TMS (×10^{-2})	Urinary	0.846	1.224	2.59 (0.75–3.71)	1.43 (0–2.96)	1.39 (0.85–2.80)	0.39	0.518
N-Acetylmannosamine-meto-4TMS (×10^{-2})	Urinary	0.808	0.661	4.26 (2.24–6.18)	3.53 (2.50–4.32)	3.14 (1.59–4.90)	0.682	0.716
N-Acetylneuraminic acid-6TMS (×10^{-1})	Urinary	0.794	0.63	1.62 (0.77–2.51)	1.25 (0.90–1.81)	0.98 (0.57–1.65)	0.979	0.413
N-Acetylserine-2TMS (×10^{-2})	Urinary	0.49	0.631	1.15 (0–1.24)	0.42 (0–1.09)	0.83 (0–1.16)	0.594	0.624
N-Acetyltyrosine-2TMS	Urinary	1.052	0.598	0 (0–0)	0 (0–0)	0 (0–0)	0.483	0.706
Niacinamide-TMS (×10^{-1})	Urinary	0.87	0.637	0.65 (0.43–1.31)	0.89 (0.49–1.50)	0.75 (0.35–1.01)	0.606	0.999
Nicotinic acid-TMS (×10^{-2})	Urinary	2.023	2.303	0.99 (0.35–1.37)	3.58 (1.57–7.82)	6.45 (2.19–10.18)	**0.031**	**0.001**
Nonanoic acid-TMS (×10^{-1})	Urinary	1.257	1.255	3.10 (2.53–3.65)	3.53 (2.88–4.15)	3.50 (2.97–3.98)	0.337	0.176
Norepinephrine-5TMS (×10^{-1})	Urinary	0.804	0.396	1.18 (0.58–2.20)	1.15 (0.82–1.41)	1.58 (0.68–2.18)	0.999	0.716
Norvaline-2TMS (×10^{-2})	Urinary	0.368	0.796	0 (0–1.01)	0 (0–1.55)	0 (0–0.80)	0.987	0.852
Octadecanol-TMS (×10^{-1})	Urinary	0.41	1.224	2.24 (2.02–2.98)	2.33 (1.88–3.00)	2.04 (1.91–2.38)	0.998	0.217
Octanoic acid-TMS (×10^{-2})	Urinary	0.772	0.603	5.34 (4.88–7.17)	6.63 (5.14–7.65)	5.85 (5.03–7.18)	0.719	0.716
Oleamide-TMS (×10^{-1})	Urinary	0.904	0.43	2.54 (1.99–4.26)	3.01 (1.83–4.38)	2.40 (1.40–3.21)	0.998	0.52
Oleic acid-TMS (×10^{-1})	Urinary	0.94	0.4	1.22 (1.09–1.35)	0.98 (0.59–1.51)	0.95 (0.77–1.76)	0.157	0.52
O-Phosphoethanolamine-4TMS	Urinary	0.723	0.849	0 (0–0)	0 (0–0)	0 (0–0)	0.808	0.768
Ornithine-4TMS (×10^{-1})	Urinary	1.355	0.761	4.07 (2.01–4.89)	4.34 (2.56–6.78)	2.39 (1.44–5.05)	0.428	0.677
Oxalic acid-2TMS (×10^{-1})	Urinary	0.935	0.951	3.75 (2.65–4.06)	3.78 (3.06–4.63)	4.02 (3.04–4.19)	0.682	0.413
Palmitic acid-TMS	Urinary	0.442	1.021	3.32 (3.03–4.19)	3.37 (2.52–4.22)	3.04 (2.73–3.73)	0.827	0.264
Palmitoleic acid-TMS (×10^{-2})	Urinary	1.027	0.671	1.57 (0.76–2.11)	0.80 (0.26–1.63)	1.24 (0.91–2.87)	0.394	0.999
Pantothenic acid-3TMS (×10^{-2})	Urinary	0.487	0.889	1.51 (0.60–2.83)	0.79 (0–3.07)	1.09 (0–1.42)	0.66	0.497
ParaXanthine-TMS (×10^{-2})	Urinary	2.003	1.43	1.53 (0.33–3.38)	4.67 (2.68–9.04)	4.53 (1.64–8.11)	**0.018**	0.155
Phenylalanine-2TMS	Urinary	0.909	1.219	1.79 (1.28–2.10)	1.44 (0.78–1.88)	0.82 (0.66–1.89)	0.428	0.24
Phenylpyruvic acid-meto-TMS	Urinary	0	0.803	0 (0–0)	0 (0–0)	0 (0–0)	0.999	0.706
Phosphoric acid-3TMS	Urinary	0.982	0.412	120.0 (94.3–175.1)	152.4 (104.1–184.4)	130.6 (99.8–158.7)	0.606	0.999
Pimelic acid-2TMS (×10^{-2})	Urinary	0.908	0.864	0 (0–0)	0 (0–1.03)	0 (0–0.54)	0.511	0.418
Proline-2TMS (×10^{-2})	Urinary	0.882	0.68	4.53 (1.85–6.15)	5.17 (2.68–6.44)	3.20 (1.72–6.76)	0.756	0.923
Protocatechuic acid-3TMS (×10^{-3})	Urinary	1.11	1.529	0 (0–2.57)	0 (0–0)	0 (0–0)	0.325	0.106
Psicose-meto-5TMS (×10^{-1})	Urinary	1.242	1.545	2.74 (0.49–1.07)	1.59 (12.6–5.47)	1.34 (0.73–2.94)	0.918	0.637

Putrescine-4TMS (×10⁻¹)	Urinary	1.108	0.524	1.25 (0.64–1.93)	1.36 (0.97–1.99)	1.42 (0.85–1.93)	0.682	0.984
Pyridoxal-meto-2TMS	Urinary	1.221	0.505	0 (0–0)	0 (0–0)	0 (0–0)	0.861	0.928
Pyrogallol-3TMS (×10⁻¹)	Urinary	1.285	1.33	0.78 (0.32–11.28)	0.98 (0.30–3.58)	0.52 (0.12–1.33)	0.999	0.412
Pyruvic acid-meto-TMS (×10⁻¹)	Urinary	0.861	0.605	2.90 (1.62–3.89)	3.22 (2.32)	3.01 (2.10–3.67)	0.942	0.984
Quinolinic acid-2TMS (×10⁻¹)	Urinary	1.222	0.962	4.18 (2.30–6.91)	3.64 (2.36–3.85)	2.91 (2.22–5.07)	0.644	0.677
Ribitol-5TMS (×10⁻¹)	Urinary	0.856	0.645	1.02 (0.66–1.56)	1.06 (0.53–1.42)	0.61 (0.41–0.99)	0.99	0.195
Ribonic acid-5TMS	Urinary	0.711	0.625	1.70 (1.07–2.38)	1.47 (1.24–2.10)	1.08 (0.72–1.56)	0.606	0.24
Ribonolactone-3TMS (×10⁻²)	Urinary	1.036	0.724	6.60 (4.05–9.89)	5.81 (3.37–8.16)	5.14 (2.94–8.51)	0.756	0.83
Ribose 5-phosphate-meto-5TMS (×10⁻³)	Urinary	0.94	0.575	0 (0–8.05)	0 (0–2.90)	0 (0–0)	0.55	0.26
Ribose-meto-4TMS (×10⁻¹)	Urinary	0.661	0.902	0.91 (0.55–1.51)	1.00 (0.67–1.25)	0.69 (0.54–0.95)	0.979	0.291
Ribulose-meto-4TMS (×10⁻¹)	Urinary	0.687	0.958	2.23 (0.97–3.25)	1.99 (1.19–3.25)	1.31 (0.93–2.03)	0.979	0.52
Saccharopine-4TMS	Urinary	0	0.803	0 (0–0)	0 (0–0)	0 (0–0)	0.999	0.706
Sebacic acid-2TMS	Urinary	1.047	0.862	0 (0–0)	0 (0–0)	0 (0–0)	0.719	0.463
Serine-3TMS	Urinary	0.871	1.205	1.73 (1.46–3.64)	1.78 (1.14–3.57)	1.24 (0.67–2.29)	0.827	0.264
Sorbitol-6TMS (×10⁻¹)	Urinary	1.121	0.609	4.53 (3.23–7.23)	4.53 (3.23–7.23)	3.27 (2.49–3.79)	0.962	**0.029**
Sorbose-meto-5TMS (×10⁻²)	Urinary	0.347	1.493	3.87 (1.89–12.82)	3.82 (2.43–9.62)	2.78 (1.32–5.78)	0.962	0.319
Spermidine-5TMS (×10⁻³)	Urinary	0.657	0.159	0 (0–8.54)	0 (0–0)	0 (0–0)	0.78	0.995
Spermine-6TMS (×10⁻¹)	Urinary	0.813	0.679	1.04 (0.64–1.35)	1.14 (0.48–1.36)	0.80 (0.38–1.64)	0.999	0.558
Stearic acid-TMS	Urinary	0.448	0.509	2.45 (2.40–3.20)	2.62 (2.26–3.17)	2.50 (2.17–3.36)	0.999	0.994
Suberic acid-2TMS (×10⁻²)	Urinary	1.089	0.912	0 (0–0.46)	0.73 (0–3.06)	0.90 (0–1.88)	0.341	0.145
Succinic acid-2TMS (×10⁻¹)	Urinary	1.239	0.238	0.95 (0.75–1.28)	0.91 (0.73–1.09)	0.95 (0.65–1.17)	0.827	0.923
Sucrose-8TMS	Urinary	2.17	2.131	1.08 (0.04–1.20)	0.17 (0.14–0.37)	0.19 (0.09–0.27)	0.428	0.52
Tagatose-meto-5TMS (×10⁻¹)	Urinary	1.299	1.641	0.93 (0.10–1.37)	0.37 (0.16–1.37)	0.27 (0.18–0.67)	0.719	0.447
Tartaric acid-4TMS	Urinary	0.639	0.646	0.12 (0.02–1.01)	0.15 (0.06–1.31)	0.13 (0.08–0.54)	0.719	0.984
Taurine-13C2-3TMS (×10⁻¹)	Urinary	0.376	0.56	0.49 (0.23–0.88)	0.68 (0.25–1.19)	0.51 (0.31–1.12)	0.827	0.793
Thiodiglycolic acid-2TMS (×10⁻³)	Urinary	1.475	0.784	0 (0–6.60)	0 (0–0)	0 (0–0)	0.21	0.148
Threitol-4TMS (×10⁻¹)	Urinary	0.948	1.058	1.25 (0.53–2.51)	1.71 (1.12–2.17)	1.07 (0.66–1.38)	0.644	0.598
Threonic acid-4TMS	Urinary	0.762	0.749	2.04 (1.01–2.54)	1.53 (1.03–2.21)	1.24 (0.79–2.26)	0.606	0.598
Threonine-3TMS (×10⁻¹)	Urinary	0.831	0.883	3.56 (2..69–5.23)	3.31 (2.21–0.601)	2.67 (1.40–4.45)	0.998	0.291

Tiglylglycine-TMS	Urinary	0.675	0.419	0 (0–0)	0 (0–0)	0 (0–0)	0.861	0.928
Trehalose-8TMS (×10^{-2})	Urinary	1.281	0.999	1.48 (0–16.58)	2.33 (0–6.08)	2.07 (1.29–7.76)	0.978	0.947
Triethanolamine-3TMS (×10^{-2})	Urinary	0.631	1.071	0 (0–1.51)	0 (0–2.90)	0 (0–0)	0.669	0.617
Tryptophan-3TMS (×10^{-1})	Urinary	1.07	0.597	1.47 (1.14–1.73)	1.87 (0.93–2.30)	0.88 (0.54–2.57)	0.532	0.348
Tyramine-3TMS (×10^{-1})	Urinary	1.111	1.738	3.74 (1.94–7.25)	2.96 (1.77–4.60)	1.85 (1.12–3.42)	0.682	0.099
Tyrosine-3TMS	Urinary	0.671	0.783	3.92 (2.29–5.77)	3.44 (1.77–5.18)	1.90 (1.27–5.66)	0.86	0.291
Uracil-2TMS (×10^{-2})	Urinary	1.013	1.56	0.59 (0–1.71)	0.58 (0–1.02)	0 (0–0.67)	0.836	0.154
Urea-2TMS	Urinary	0.708	0.503	172.1 (150.3–237.0)	201.6 (169.0–250.2)	197.6 (144.0–263.1)	0.86	0.968
Ureidopropionic acid-2TMS (×10^{-3})	Urinary	0.643	0.498	8.46 (0–9.92)	0 (0–4.31)	0 (0–10.97)	0.252	0.799
Uric acid-4TMS	Urinary	0.727	0.446	4.48 (3.00–7.53)	3.59 (3.12–4.86)	4.70 (3.00–10.66)	0.644	0.948
Urocanic acid-2TMS (×10^{-1})	Urinary	0.428	1.302	2.08 (1.03–2.54)	1.52 (1.09–1.97)	1.12 (0.88–1.47)	0.644	0.141
Valine-2TMS	Urinary	0.72	1.151	1.64 (1.11–1.91)	1.52 (0.85–1.80)	0.97 (0.71–1.72)	0.89	0.291
Vanilmandelic acid-3TMS (×10^{-1})	Urinary	0.836	0.599	5.52 (2.29–8.37)	1.98 (2.93–6.07)	4.47 (2.66–5.57)	0.756	0.716
Xanthine-3TMS (×10^{-1})	Urinary	0.887	0.611	0.54 (0.37–1.33)	0.66 (0.37–1.24)	0.39 (0.29–0.80)	0.918	0.558
Xanthosine-5TMS (×10^{-2})	Urinary	0.975	0.663	1.08 (0.27–1.43)	1.03 (0–1.69)	0.49 (0–1.14)	0.998	0.432
Xylitol-5TMS (×10^{-1})	Urinary	1.039	0.663	1.37 (0.64–2.22)	1.12 (0.80–1.74)	0.90 (0.61–1.42)	0.998	0.483
Xylose-meto-4TMS (×10^{-1})	Urinary	1.525	0.661	1.20 (0.36–1.65)	1.45 (0.89–2.74)	0.80 (0.49–1.58)	0.283	0.968
Xylulose-meto-4TMS (×10^{-1})	Urinary	0.712	1.014	3.05 (1.05–3.57)	2.48 (1.64–3.28)	1.66 (1.24–2.37)	0.979	0.558

Abbreviations: IQR, Interquartile range; arb.unit, arbitrary unit; VIP, variable importance for projection; AHU, asymptomatic hyperuricemia; SUA, serum urate level; TMS, tetramethylsilane. VIP [1] value was obtained from PLS regression analysis distinguished gout with SUA <9.0 mg/dL from asymptomatic hyperuricemia. VIP [2] value was obtained from PLS regression analysis distinguished gout with SUA ≥9.0 mg/dL from asymptomatic hyperuricemia. Raw P [3] and P [4] values were calculated from Steel with control tests comparing asymptomatic hyperuricemia to gout with SUA <9.0 mg/dL, and asymptomatic hyperuricemia to gout with SUA ≥9.0 mg/dL, respectively (without the Benjamini-Hochberg adjustment).

Table S3. Summary of pathway analysis using potential biomarkers distinguishing overall gout and asymptomatic hyperuricemia groups.

**Metabolism**	**Raw p**	**$-log_{10}(p)$**	**Adjusted *p***	**$-log_{10}$ (adjusted p)**	**Impact**
Citrate cycle (TCA cycle)	3.33×10^{-5}	4.477	0.003	2.553	0.200
Glycolysis / Gluconeogenesis	0.002	2.692	0.057	1.247	0.101
Alanine, aspartate and glutamate metabolism	0.003	2.597	0.057	1.247	0.135
Taurine and hypotaurine metabolism	0.003	2.569	0.057	1.247	0.286
Glyoxylate and dicarboxylate metabolism	0.004	2.428	0.063	1.203	0.024
Butanoate metabolism	0.010	2.013	0.136	0.867	0.032
Pyruvate metabolism	0.020	1.689	0.246	0.610	0.207
Glutathione metabolism	0.032	1.491	0.339	0.470	0.011
Glycine, serine and threonine metabolism	0.044	1.359	0.368	0.434	0.000
Cysteine and methionine metabolism	0.044	1.359	0.368	0.434	0.096
Biosynthesis of unsaturated fatty acids	0.051	1.290	0.391	0.408	0.000
Arginine and proline metabolism	0.057	1.247	0.391	0.408	0.024
D-Glutamine and D-glutamate metabolism	0.060	1.219	0.391	0.408	0.000
Thiamine metabolism	0.070	1.154	0.421	0.376	0.000
Aminoacyl-tRNA biosynthesis	0.086	1.068	0.479	0.320	0.000
Caffeine metabolism	0.099	1.005	0.519	0.285	0.692
Arginine biosynthesis	0.136	0.867	0.671	0.173	0.000
Nicotinate and nicotinamide metabolism	0.145	0.839	0.675	0.170	0.000
Starch and sucrose metabolism	0.171	0.766	0.756	0.122	0.050
Pantothenate and CoA biosynthesis	0.180	0.745	0.756	0.122	0.000

Pathway analysis of the potential biomarkers was applied within the MetaboAnalyst 5.0 (https://www.metaboanalyst.ca). Pathway analysis was performed using referred KEGG (http://www.kegg.jp) homo sapiens pathway library and using the Hypergeometric test. P-values were adjusted by the Benjamini-Hochberg method to control the false discovery rate (FDR).

Table S4. Summary of pathway analysis using potential biomarkers distinguishing gout with SUA ≥9.0 mg/dL and asymptomatic hyperuricemia groups.

Metabolism	Raw p	$-log_{10}(p)$	Adjusted *p*	$-log_{10}$ (adjusted p)	Impact
Citrate cycle (TCA cycle)	1.38×10^{-6}	5.861	1.16×10^{-4}	3.937	0.512
Glyoxylate and dicarboxylate metabolism	0.000	3.816	0.006	2.223	0.182
Alanine, aspartate and glutamate metabolism	0.000	3.670	0.006	2.223	0.333
Pyruvate metabolism	0.001	2.832	0.031	1.509	0.455
Glycolysis or Gluconeogenesis	0.004	2.449	0.060	1.224	0.200
Arginine biosynthesis	0.009	2.069	0.118	0.929	0.115
Butanoate metabolism	0.010	2.008	0.118	0.929	0.143
Nicotinate and nicotinamide metabolism	0.020	1.699	0.210	0.678	0.122
Biosynthesis of unsaturated fatty acids	0.025	1.606	0.231	0.636	0.043
Glycine, serine and threonine metabolism	0.049	1.310	0.398	0.400	0.045
D-Glutamine and D-glutamate metabolism	0.052	1.283	0.398	0.400	0.222
Arginine and proline metabolism	0.063	1.203	0.439	0.358	0.091
Histidine metabolism	0.158	0.801	1.000	0.000	0.032
Pantothenate and CoA biosynthesis	0.167	0.777	1.000	0.000	0.030
beta-Alanine metabolism	0.211	0.675	1.000	0.000	0.047
Porphyrin and chlorophyll metabolism	0.249	0.604	1.000	0.000	0.038
Cysteine and methionine metabolism	0.319	0.496	1.000	0.000	0.071
Aminoacyl-tRNA biosynthesis	0.331	0.481	1.000	0.000	0.014
Arachidonic acid metabolism	0.356	0.449	1.000	0.000	0.275
Tyrosine metabolism	0.380	0.420	1.000	0.000	0.011

Pathway analysis of the potential biomarkers was applied within the MetaboAnalyst 5.0 (https://www.metaboanalyst.ca). Pathway analysis was performed using referred KEGG (http://www.kegg.jp) homo sapiens pathway library and using the Hypergeometric test. P-values were adjusted by the Benjamini-Hochberg method to control the false discovery rate (FDR).
